# Unveiling novel cell clusters and biomarkers in glioblastoma and its peritumoral microenvironment at the single-cell perspective

**DOI:** 10.1186/s12967-024-05313-5

**Published:** 2024-06-08

**Authors:** Liping Wang, Xinyi Li, Chengshi Xu, Danwen Wang, Chao Ma, Zefen Wang, Yirong Li, Zhiqiang Li

**Affiliations:** 1 Department of Laboratory Medicine, Zhongnan Hospital of Wuhan University, Wuhan University, Wuhan, 430071 Hubei China; 2 Department of Neurosurgery, Zhongnan Hospital of Wuhan University, Wuhan University, Wuhan, 430071 Hubei China; 3 Department of Physiology, Wuhan University School of Basic Medical Sciences, Wuhan University, Wuhan, 430071 Hubei China; 4 Brain Glioma Center, Zhongnan Hospital of Wuhan University, Wuhan University, Wuhan, 430071 Hubei China

**Keywords:** Biomarkers, Glioblastoma, Tumor microenvironment, Tumor heterogeneity, Single-cell sequencing

## Abstract

**Background:**

Glioblastoma (GBM) is a highly heterogeneous, recurrent and aggressively invasive primary malignant brain tumor. The heterogeneity of GBM results in poor targeted therapy. Therefore, the aim of this study is to depict the cellular landscape of GBM and its peritumor from a single-cell perspective. Discovering new cell subtypes and biomarkers, and providing a theoretical basis for precision therapy.

**Methods:**

We collected 8 tissue samples from 4 GBM patients to perform 10 × single-cell transcriptome sequencing. Quality control and filtering of data by Seurat package for clustering. Inferring copy number variations to identify malignant cells via the infercnv package. Functional enrichment analysis was performed by GSVA and clusterProfiler packages. STRING database and Cytoscape software were used to construct protein interaction networks. Inferring transcription factors by pySCENIC. Building cell differentiation trajectories via the monocle package. To infer intercellular communication networks by CellPhoneDB software.

**Results:**

We observed that the tumor microenvironment (TME) varies among different locations and different GBM patients. We identified a proliferative cluster of oligodendrocytes with high expression of mitochondrial genes. We also identified two clusters of myeloid cells, one primarily located in the peritumor exhibiting an M1 phenotype with elevated *TNFAIP8L3* expression, and another in the tumor and peritumor showing a proliferative tendency towards an M2 phenotype with increased *DTL* expression. We identified *XIST*, *KCNH7*, *SYT1* and *DIAPH3* as potential factors associated with the proliferation of malignant cells in GBM.

**Conclusions:**

These biomarkers and cell clusters we discovered may serve as targets for treatment. Targeted drugs developed against these biomarkers and cell clusters may enhance treatment efficacy, optimize immune therapy strategies, and improve the response rates of GBM patients to immunotherapy. Our findings provide a theoretical basis for the development of individualized treatment and precision medicine for GBM, which may be used to improve the survival of GBM patients.

**Supplementary Information:**

The online version contains supplementary material available at 10.1186/s12967-024-05313-5.

## Introduction

Glioblastoma (GBM) is an invasive tumor with a high recurrence rate, accounting for approximately 48% of all primary central nervous system malignancies [1, 2]. Although various treatment methods for glioblastoma have made progress in recent years, the overall prognosis is poor, with a median overall survival (OS) of less than 15 months, and 90% of cases are estimated to relapse within 12 months after diagnosis [1, 3].

Glioblastoma has a highly complex and heterogeneous tumor microenvironment (TME), which is composed of stromal cells, signaling molecules, immune cells, and the surrounding extracellular matrix [4]. The TME affects the behavior and progression of GBM cells, mainly inducing tumor invasiveness and drug resistance. Immunosuppressive TME provides multiple pathways for tumor immune escape [5].

Mitochondria are crucial organelles responsible for many physiological processes, including cell metabolism, reactive oxygen species production, and play a role in cell death signaling, innate immunity and autophagy. Mitochondrial dysfunction is also an important cause of cell death or failure, the excess of reactive oxygen species cause DNA damage and other damage to cells, and the mutations in cellular metabolism genes may trigger cancer initiation [6–8]. Previous studies have observed mutations in the mtDNA gene in various cancer cells, mitochondrial alterations may promote cancer progression through Warburg effect and metabolic reprogramming [9, 10]. In addition, changes in energy metabolism or mitochondria may inhibit the anticancer function of immune cells and enhance the immune escape of cancer cells in the TME [11].

Infiltration diffusion is a characteristic of glioblastoma, and it is also an important cause of widespread recurrence in glioblastomas patients [12]. The recurrence after total surgical resection and adjuvant chemotherapy almost always occurs in the so-called peritumoral brain zone (PBZ). PBZ is more like a transitional zone with a precancerous microenvironment, which is the basis for glioblastoma progression and recurrence. The understanding of PBZ may be related to the development of more effective treatment methods to prevent the development and recurrence of glioblastoma [13].

In this study, to determine cell diversity within the tumor core and surrounding brain, we performed single-cell sequencing on clinical samples from four patients with primary glioblastoma, each collecting samples from two different locations: the first located within the tumor core and the second from the peritumoral brain. We sequenced a total of 67875 cells, including tumor cells, myeloid cells, lymphocytes, neurons and oligodendrocytes. Our data provides a large-scale dissection of glioblastoma cell types and their respective gene expression profiles, revealing a wealth of information on the interactions between tumor cells and the immune system. These observations will help us better understand the complexity of the immune microenvironment in the tumor center and peritumoral brain zone, and provide a basis for the development of new immune and targeted therapies.

## Methods

### Sample collection, library construction and sequencing

We collected tumor and peritumoral tissue samples from 4 untreated glioblastoma patients between September 2021 and November 2022 at the Department of Neurosurgery, Zhongnan Hospital of Wuhan University. The study was approved by the Ethics Committee, Zhongnan Hospital of Wuhan University (ethics No. 2019048). A total of 8 tissue samples were subjected to 10 × single-cell sequencing. The nucleus suspension, with a concentration ranging from 700 to 1200 per microliter as determined by Count Star, was loaded onto the Chromium Single Cell Controller (10 × Genomics) to generate single-cell gel beads in emulsion (GEMs) following the manufacturer's protocol, using the Single Cell 3' Library and Gel Bead Kit v3.1 (10 × Genomics, 1000268) and Chromium Single Cell G Chip Kit (10 × Genomics, 1000120). Single nuclei were suspended in PBS containing 0.04% BSA. Approximately 20,000 nuclei were loaded into each channel, with an estimated recovery target of about 10,000 cells. The captured cells underwent lysis, and the released RNA was barcoded through reverse transcription within individual GEMs. Following the manufacturer's guidelines, Single-cell RNA-seq libraries were generated using the Single Cell 3' Library and Gel Bead Kit v3.1. The sequencing of libraries was conducted on an Illumina Novaseq 6000 sequencer, ensuring a sequencing depth of a minimum of 40,000 reads per cell with a paired-end 150 bp (PE150) reading strategy. This sequencing process was carried out by CapitalBio Technology, Beijing.

### Data quality control

Fastq files were processed to generate a feature-barcode matrix using the cellranger count module. The Seurat package (v4.2.0) was employed for quality control and filtering of the feature-barcode matrix. Cells with fewer than 200 genes, in the top 1% for gene count, or with a mitochondrial gene ratio over 5% were considered abnormal and filtered out. Harmony was applied to remove batch effects among samples, while PCA was employed for dimensionality reduction, and UMAP was used for visualization.

### InferCNV and annotation

We first performed unsupervised clustering of cells and then provided preliminary annotations to cell clusters based on previously reported markers, The Human Protein Atlas database (https://www.proteinatlas.org/), and the CellMarker 2.0 database (http://117.50.127.228/CellMarker/index.html) [14–19]. Selecting a reference cell cluster, we utilized the infercnv package (v1.6.0) to infer copy number variations in cells, thus identifying malignant cell clusters. Using the FindAllMarkers function and sorting based on avg_log2FC in descending order, we selected the top 20 genes as candidate marker genes for this cell subtype (p_val_adj < 0.05 and Wilcoxon test). From these 20 genes, one was chosen to represent the characteristic of this cell subtype. If all 20 genes were highly expressed in all cell subtypes, then no gene was selected to represent the feature of that cell subtype.

### Functional enrichment and protein–protein interaction

Using the HALLMARK gene set, GSVA package (v1.38.2) was employed to assess gene set enrichment results for single-cell expression profile data. Subsequently, differential analysis was performed through the limma package (v3.46.0), with p-values computed using a Bayesian approach. The FindMarkers function was utilized to obtain differentially expressed genes in cell subtypes between the tumor and peritumor (p_val_adj < 0.05, abs(avg_log2FC) > 0.4, and Wilcoxon test). The clusterProfiler package (v4.6.2) was then employed for GO and KEGG functional enrichment analysis of differentially expressed genes (P < 0.05). Furthermore, the differentially expressed genes were submitted to the STRING database (v12.0) to construct a protein–protein interaction network. For complex protein–protein interaction networks, the top 10 proteins were extracted using the cytoHubba in Cytoscape software (v3.9.1) to visualize the protein–protein interaction network.

### Module scores and transcription factor predictions

We calculated scores for myeloid cell subgroups M1 and M2 using the markers through the AddModuleScore function. Subsequently, we compared scores of myeloid cell subgroups M1 and M2 using the Wilcoxon test. We utilized pySCENIC (v0.10.3) to compute co-expression among genes based on the gene expression matrix. Using motif data for transcription factors, we predicted and filtered co-expression relationships between transcription factors and target genes, obtaining cell-specific transcriptional regulatory modules.

### Cell trajectory inference and intercellular communication

We utilized monocle2 (v2.4.0) to select feature genes, performed dimensionality reduction based on the selected genes (q-value < 0.01). Then, a graph was constructed using machine learning and cell ordering to establish the trajectory of cell differentiation. We also utilized velocyto.R (v0.6) to infer dynamic changes in cell states by estimating the abundance of unspliced and spliced mRNA over time. The CellPhoneDB software (v2.0) utilizes gene expression matrices and cell clustering to obtain the average expression and significant relationships of ligand-receptor complexes, thereby inferring interactions between cell clusters. We also employed the iTALK package (v0.1.0) to visualize the interaction network between cell clusters.

## Results

### Preliminary exploration of the GBM microenvironment

**Fig. 1:**
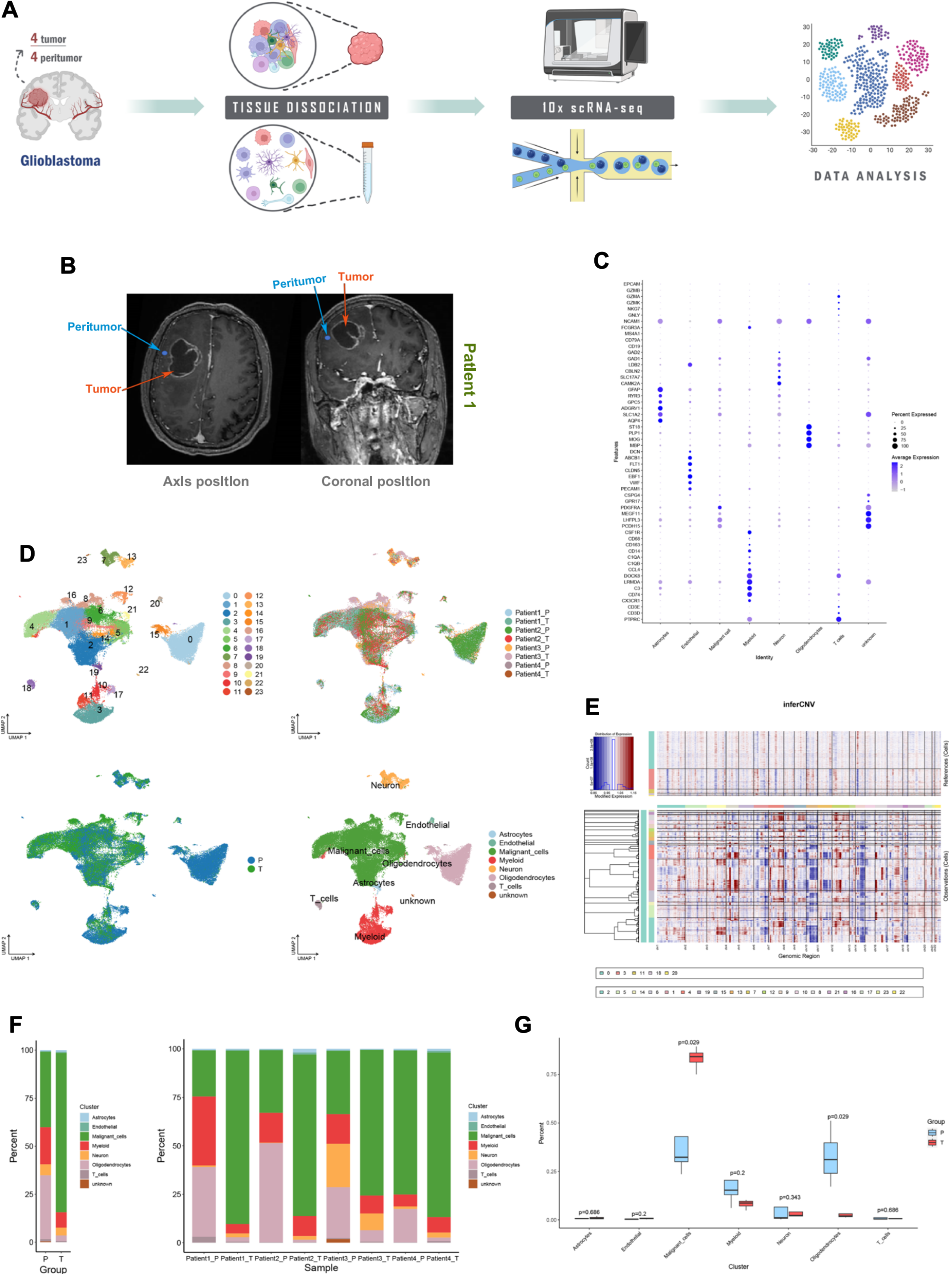
Preliminary exploration of the GBM microenvironment. **A** Flowchart. **B** Magnetic Resonance Imaging in Patient 1. **C** Biological markers for each cell cluster. **D** The uniform manifold approximation and projection (UMAP) plots of cell clusters in GBM, 24 clusters (top left), patient origin (top right), site of origin (bottom left), annotated cell subgroups (bottom right). Each point represents a cell, with different colors indicating different cell clusters, patients, and origins. **E** inferCNV, where red represents copy number gain, and blue represents copy number loss. **F** Percentage distribution of cell clusters after annotation, with different colors representing different cell clusters. **G** Differences in the content of cell clusters between the tumor and its surrounding area

We collected 4 tumor samples and 4 peritumoral samples from 4 GBM patients for 10 × single-cell sequencing (Fig. 1A, B and Table 1). We obtained a total of 67,875 cells, with a median UMI count of 5,635 per cell and a median gene count of 2721 per cell (Fig. s1A). After data quality control and filtering, 63,998 cells were used for subsequent data analysis. The distribution of data for each sample after quality control is shown in Supplementary Fig. 1B. Through unsupervised clustering, the 63,998 cells were grouped into 24 clusters. Based on previously reported biomarkers, The Human Protein Atlas database, CellMarker 2.0 database and copy number variations, these 24 clusters were further categorized into Astrocytes, Endothelial, Malignant cells, Myeloid, Neuron, Oligodendrocytes, and T cells. Heterogeneity was observed among these cell clusters across different regions and patients (Fig. 1C, D and E). Cluster 0 predominantly occupies the peritumor, while Cluster 1 is most abundant within the tumor. Clusters 8 and 16 are primarily found in the tumors of Patient 3 (Fig. s1C). Malignant cells, Myeloid, and Oligodendrocytes constitute the main components in the peritumor. Malignant cells have the highest proportion within the tumor. T cells are predominantly present in the peritumor of Patient 1 (Fig. 1F). Malignant cells (P = 0.029) exhibit a higher abundance within the tumor compared to the peritumor. Oligodendrocytes (P = 0.029) show a higher abundance in the peritumor than within the tumor. The abundance of Myeloid cells is higher in the peritumor compared to within the tumor; however, the difference is not statistically significant (Fig. 1G). In the GBM microenvironment, there are Astrocytes, Endothelial cells, Malignant cells, Myeloid cells, Neurons, Oligodendrocytes and T cells. The predominant immune cells in the GBM microenvironment are Myeloid cells. Compared to the tumor, the peritumor contains a large number of Myeloid and Oligodendrocytes. The composition and abundance of these cell clusters vary in the GBM microenvironment among different patients.

**Table 1:** Clinical information of patients

Patients	Age	Gender	Location	IDH mutational status	ATRX	1p19q codeletion	MGMT methylation
Patient 1	64	Male	Right frontal lobe	WT	WT	NO	Methylated
Patient 2	62	Female	Left temporal occipital lobe	WT	WT	NO	Methylated
Patient 3	58	Female	Right frontal lobe	WT	WT	NO	Low-level methylated
Patient 4	52	Male	Left frontal lobe	WT	WT	NO	Low-level methylated

### The role of subtypes of oligodendrocytes in the GBM microenvironment

**Fig. 2:**
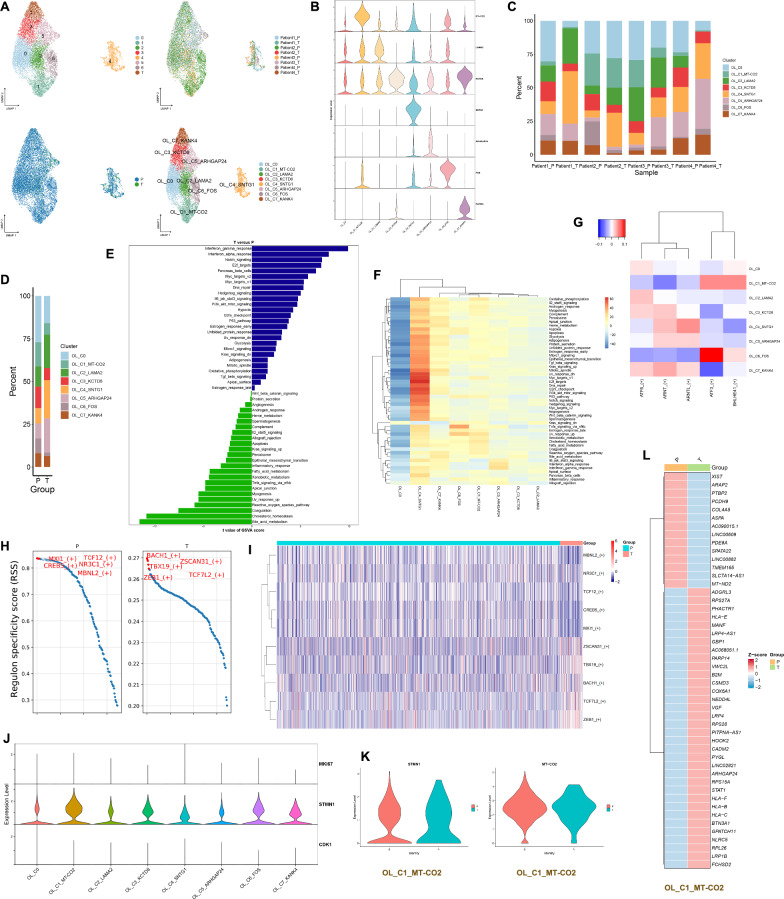
The role of subtypes of oligodendrocytes in the GBM microenvironment. **A** UMAP plots of oligodendrocyte cell clusters in the GBM microenvironment, 8 clusters (top left), patient origin (top right), site of origin (bottom left), annotated cell subgroups (bottom right). Each point represents a cell, with different colors indicating different cell clusters, patients, and origins. **B** One gene from the top 20 genes in each cell cluster is selected as a biomarker for that specific cell cluster. **C** and **D** Percentage distribution of cell clusters after annotation, with different colors representing different cell clusters. **E** Gene set variation analysis (GSVA) in oligodendrocytes (tumor versus peritumor). **F** GSVA, a heatmap showing pathway enrichment for each cell cluster. **G** SCENIC, a transcription factor heatmap for each cell cluster. **H** and **I** Top 5 transcription factors for both tumor and peritumor. **J** The expression of proliferative genes in each cell cluster. (K) Expression levels of *STMN1* and *MT-CO2* in OL_C1_MT-CO2 in the tumor and peritumor. **L** Differential analysis in OL_C1_MT-CO2 using Wilcoxon test, with avg_log2FC > 0.4 and p_val_adj < 0.05. Red indicates high expression, while blue indicates low expression

**Fig. 3:**
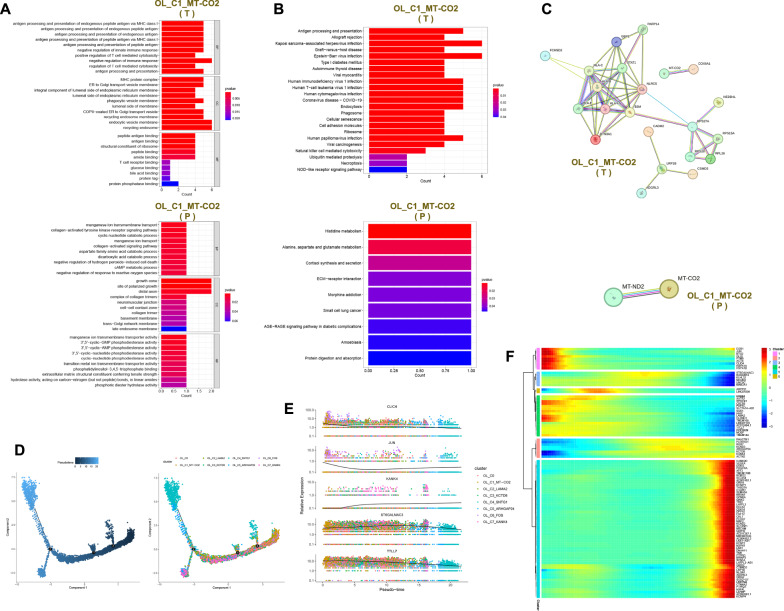
Functional enrichment and cell trajectory inference in oligodendrocytes. **A** GO functional enrichment of OL_C1_MT-CO2 in the tumor and peritumor. **B** KEGG functional enrichment of OL_C1_MT-CO2 in the tumor and peritumor. **C** Protein–protein interaction network of OL_C1_MT-CO2 in the tumor and peritumor. **D** Cell trajectory inference, with each point representing a cell. The gradient from deep blue to light blue indicates time progression from early to late (left), and different colors represent different cell clusters (right). **E** Dynamic expression of top 5 genes in oligodendrocyte cell clusters, with different colors representing different clusters. **F** Dynamic expression of top 100 genes in oligodendrocyte cell clusters, with a gradient from blue to red indicating expression levels from low to high

The 11,930 oligodendrocyte cells were grouped into 8 clusters, and the patient and site of origin for each cluster are shown in Fig. 2A. One gene was selected to represent the characteristic features of each cell cluster from the top 20 genes identified through differential analysis for each cell cluster (Fig. 2B and Table S1). These eight cell clusters were ultimately annotated as OL_C0, OL_C1_MT-CO2, OL_C2_LAMA2, OL_C3_KCTD8, OL_C4_SNTG1, OL_C5_ARHGAP24, OL_C6_FOS and OL_C7_KANK4 (Fig. 2A). OL_C6_FOS is mainly present in the peritumor of Patient 2 and is absent in the tumor of Patient 1. OL_C2_LAMA2 is absent in the tumor of Patient 4 (Fig. 2C). OL_C2_LAMA2, OL_C4_SNTG1 and OL_C5_ARHGAP24 are the main components of the tumor (Fig. 2D and Fig. s1D). These oligodendrocytes are mainly enriched in pathways such as interferon gamma response, interferon alpha response and notch signaling in the tumor, while in the peritumor, they are primarily enriched in pathways like bile acid metabolism, cholesterol homeostasis and coagulation (Fig. 2E). OL_C4_SNTG1 is primarily enriched in pathways like DNA repair, Pi3k akt mtor signaling and P53 pathway. OL_C1_MT-CO2 is mainly enriched in pathways such as oxidative phosphorylation, xenobiotic metabolism and fatty acid metabolism (Fig. 2F). *ATF3* and *BHLHE41* demonstrate elevated activity in OL_C1_MT-CO2, whereas *ATF3* exhibits high activity in OL_C6_FOS (Fig. 2G). In the tumor, the top 5 active transcription factors are *BACH1*, *ZSCAN31*, *TBX19*, *ZEB1* and *TCF7L2*. In the peritumor, the top 5 active transcription factors are *MXI1*, *TCF12*, *CREB5*, *NR3C1* and *MBNL2* (Fig. 2H and Fig. 2I). According to previously reported proliferative genes in GBM (*MKI67*, *STMN1* and *CDK1*), we observed that OL_C1_MT-CO2 exhibits high expression of *STMN1*, indicating high proliferative capacity (Fig. 2J). OL_C1_MT-CO2 expresses both *STMN1* and *MT-CO2* in both the tumor and peritumor, suggesting the presence of OL_C1_MT-CO2 in both locations (Fig. 2K). Different genes highly expressed in OL_C1_MT-CO2 in the tumor or peritumor are shown in Fig. 2L. Gene ontology (GO) enrichment analysis showed that in the tumor OL_C1_MT-CO2 is participated in biological processes such as antigen processing and presentation of endogenous peptide antigen via MHC class I, and exert functions including peptide antigen binding, antigen binding and structural constituent of ribosome. However, the OL_C1_MT-CO2 in the peritumor is involved in biological processes such as manganese ion transmembrane transport, collagen − activated tyrosine kinase receptor signaling pathway and cyclic nucleotide catabolic process, and perform functions such as manganese ion transmembrane transporter activity, 3',5' − cyclic − GMP phosphodiesterase activity and 3',5' − cyclic − AMP phosphodiesterase activity (Fig. 3A). Similarly, through Kyoto encyclopedia of genes and genomes (KEGG) enrichment analysis, we found that OL_C1_MT-CO2 is involved in antigen processing and presentation in the tumor. In the peritumor, OL_C1_MT-CO2 is associated with histidine, alanine, aspartate and glutamate metabolism (Fig. 3B). The protein–protein interaction networks of OL_C1_MT-CO2 in the tumor and peritumor are shown in Fig. 3C. According to the cell trajectory inference, the potential differentiation endpoint of oligodendrocytes might be OL_C4_SNTG1 (Fig. 3D). As oligodendrocytes differentiate, the expression of *TTLL7* gradually decreases, while the expression of *KANK4* increases (Fig. 3E). As time progresses, the expression profile of the top 100 genes in oligodendrocytes is shown in Fig. 3F. During the single-cell data analysis process, cells with high expression of mitochondrial genes are generally considered to be undergoing death. In our study, oligodendrocytes with high expression of MT-CO2 also highly express STMN1, suggesting strong proliferative capacity. oligodendrocytes with high expression of MT-CO2 are present in each patient and are predominantly present in large numbers in the peritumor. They are involved in oxidative phosphorylation, xenobiotic metabolism and fatty acid metabolism. At the protein level, MT-CO2 and COX6A1 interacted in the tumor, whereas MT-CO2 and MT-ND2 interacted in the peritumor. MT-CO2, COX6A1, and MT-ND2 may be involved in shaping the GBM microenvironment.

### The function of myeloid clusters in the GBM microenvironment

**Fig. 4:**
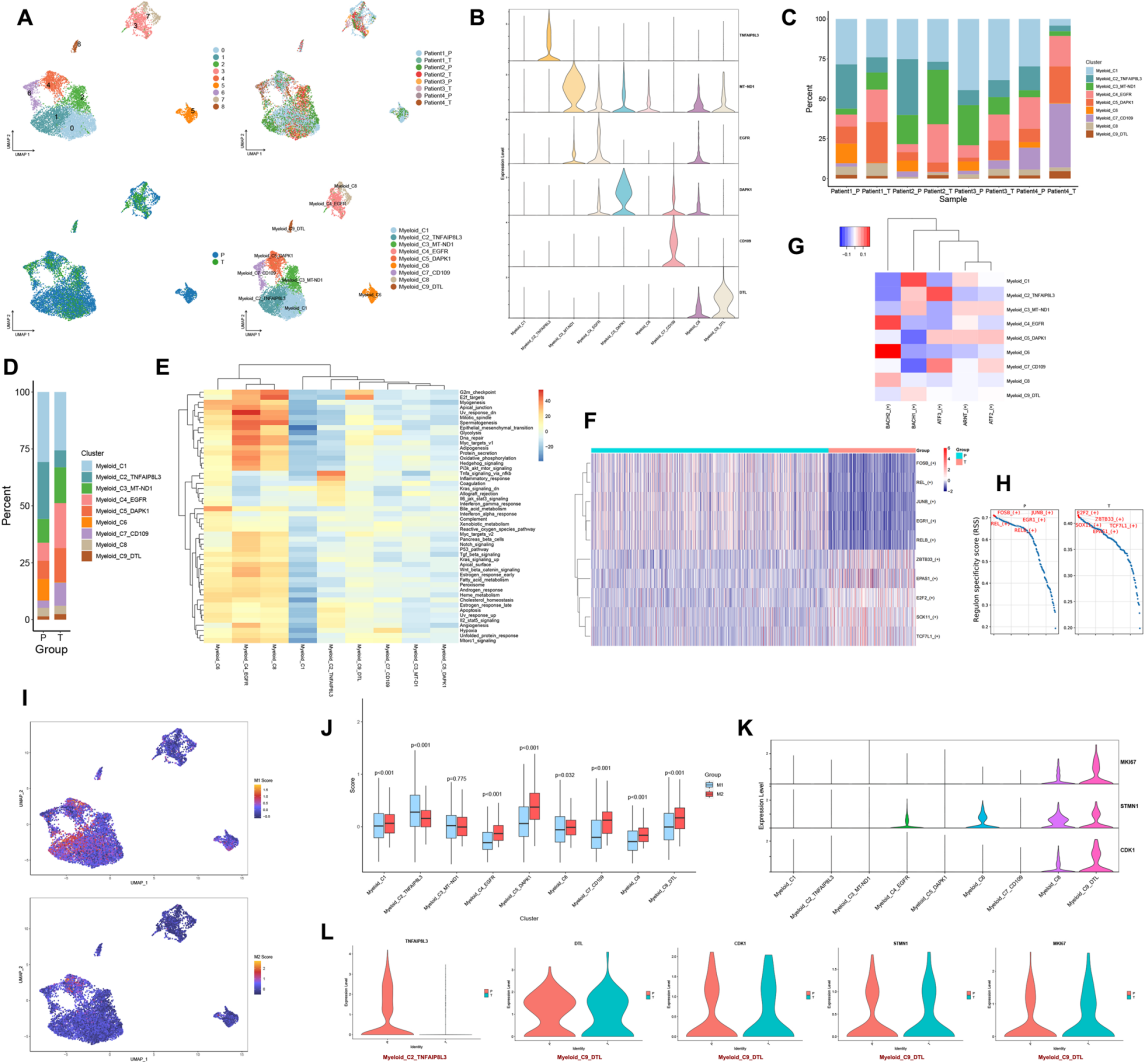
The function of myeloid clusters in the GBM microenvironment. **A** UMAP plots of myeloid cell clusters in the GBM microenvironment, 9 clusters (top left), patient origin (top right), site of origin (bottom left), annotated cell subgroups (bottom right). Each point represents a cell, with different colors indicating different cell clusters, patients, and origins. **B** One gene from the top 20 genes in each cell cluster is selected as a biomarker for that specific cell cluster. **C** and **D** Percentage distribution of cell clusters after annotation, with different colors representing different cell clusters. **E** GSVA, a heatmap showing pathway enrichment for each cell cluster. **F** and **H** Top 5 transcription factors for both tumor and peritumor. **G** SCENIC, a transcription factor heatmap for each cell cluster. **I** UMAP plots of M1 and M2 scores, with each point representing a cell. The color gradient from deep purple to yellow signifies higher scores. **J** Differences of M1 and M2 scores in different myeloid cell clusters. **K** The expression of proliferative genes in each cell cluster. **L** Expression levels of *TNFAIP8L3* in Myeloid_C2_TNFAIP8L3 in the tumor and peritumor. Expression levels of *DTL*, *CDK1*, *STMN1* and *MKI67* in Myeloid_C9_DTL in the tumor and peritumor

**Fig. 5:**
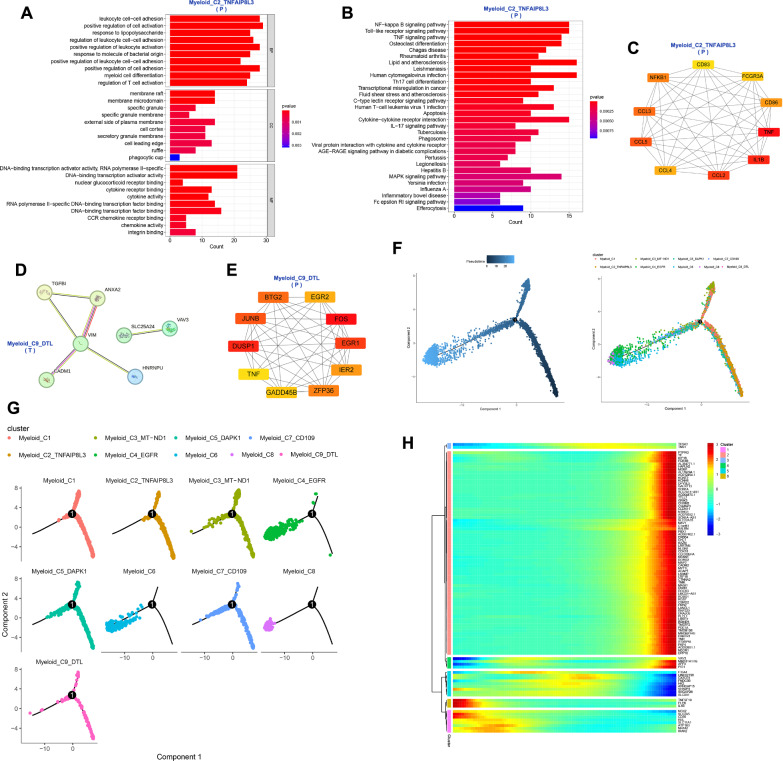
Functions and developmental trajectories of myeloid. **A** GO functional enrichment of Myeloid_C2_TNFAIP8L3 in the peritumor. **B** KEGG functional enrichment of Myeloid_C2_TNFAIP8L3 in the peritumor. **C** Protein–protein interaction network of Myeloid_C2_TNFAIP8L3 in the peritumor. **D** and **E** Protein–protein interaction networks of Myeloid_C9_DTL in the tumor and peritumor. **F** Cell trajectory inference, with each point representing a cell. The gradient from deep blue to light blue indicates time progression from early to late (left), and different colors represent different cell clusters (right). **G** The cell trajectories for each myeloid cell cluster, where different colors represent different cell clusters. **H** Dynamic expression of top 100 genes in myeloid cell clusters, with a gradient from blue to red indicating expression levels from low to high

8874 myeloid cells were divided into 9 clusters, and their patients’ and locations' sources can be seen in Fig. 4A. For each cell cluster, we selected one gene to represent its characteristics from the top 20 genes identified through differential analysis (Fig. 4B and Table S1). The 9 cell clusters were annotated as Myeloid_C1, Myeloid_C2_TNFAIP8L3, Myeloid_C3_MT-ND1, Myeloid_C4_EGFR, Myeloid_C5_DAPK1, Myeloid_C6, Myeloid_C7_CD109, Myeloid_C8 and Myeloid_C9_DTL (Fig. 4A). Myeloid_C7_CD109 is predominantly present in Patient 4 and is mainly found in the tumor (Fig. 4C). Myeloid_C2_TNFAIP8L3 and Myeloid_C6 are mainly found in the peritumor. Myeloid_C4_EGFR, Myeloid_C5_DAPK1 and Myeloid_C9_DTL have higher proportions in the tumor compared to the peritumor (Fig. 4D and Fig. s1D). The active pathways for Myeloid_C4_EGFR include epithelial-mesenchymal transition, glycolysis, DNA repair and angiogenesis. Myeloid_C2_TNFAIP8L3 is mainly involved in TNF-α signaling via NF-kB and inflammatory response. Myeloid_C9_DTL is predominantly enriched in pathways related to G2/M checkpoint and E2F targets (Fig. 4E). These myeloid cells are predominantly enriched in pathways such as oxidative phosphorylation, glycolysis and E2F targets in the tumor. In the peritumor, these myeloid cells are mainly enriched in pathways including TNF-α signaling via NF-kB, Inflammatory response and IL6 Jak-STAT3 signaling (Fig. s1E). In the tumor, the top 5 transcription factors for Myeloid are *E2F2*, *ZBTB33*, *SOX11*, *TCF7L1* and *EPAS1*. In the peritumor, the top 5 transcription factors for Myeloid are *FOSB*, *JUNB*, *EGR1*, *REL* and *RELB* (Fig. 4F and H). *ATF3* is active in Myeloid_C2_TNFAIP8L3, while *BACH2* is active in Myeloid_C4_EGFR (Fig. 4G). Based on the CellMarker 2.0 database and previous literature, we obtained markers for M1 macrophages (*FCGR3A*, *CD86*, *NOS2*, *PTGS2*, *TLR2* and *IL1B*) and M2 macrophages (*ARG1*, *CD163*, *CD68*, *MSR1*, *MRC1* and *F13A1*) [20, 21]. However, based on these markers, we cannot fully distinguish M1 and M2 macrophages in GBM (Fig. s2C). Based on these markers, we calculated the scores for M1 and M2 using the AddModuleScore function. We found that Myeloid_C2_TNFAIP8L3 (p < 0.001) tends to exhibit an M1 phenotype, while Myeloid_C9_DTL (p < 0.001) tends to exhibit an M2 phenotype (Fig. 4I and J). Myeloid_C9_DTL also exhibits high expression of *MKI67*, *STMN1* and *CDK1*, suggesting that Myeloid_C9_DTL is a highly proliferative cell population with a phenotype similar to M2 (Fig. 4K). The marker gene *TNFAIP8L3* of Myeloid_C2_TNFAIP8L3 is primarily expressed in the peritumor, while the marker gene *DTL* and proliferative genes (*MKI67*, *STMN1* and *CDK1*) of Myeloid_C9_DTL are expressed in both the tumor and peritumor (Fig. 4L). Differential genes for Myeloid_C2_TNFAIP8L3 and Myeloid_C9_DTL in tumor and peritumor can be seen in subfigures A and B. Myeloid_C2_TNFAIP8L3 in the peritumor is involved in biological processes such as leukocyte cell–cell adhesion, positive regulation of cell activation and response to lipopolysaccharide, and plays a role in DNA-binding transcription activator activity, RNA polymerase II-specific and DNA-binding transcription activator activity. It also regulates the NF-kappa B signaling pathway, Toll-like receptor signaling pathway and TNF signaling pathway (Fig. 5A, B). The top 10 protein networks of Myeloid_C2_TNFAIP8L3 in the peritumor are shown in Fig. 5C. Among these top 10 proteins are M1-type markers (*FCGR3A*, *CD86* and *IL1B*) and chemokines (*CCL2*, *CCL3*, *CCL4* and *CCL5*), further supporting the tendency of Myeloid_C2_TNFAIP8L3 toward an M1 phenotype and its potential recruitment to the TME. Myeloid_C9_DTL in the tumor participates in the biological process such as positive regulation of cell adhesion, focal adhesion assembly and cell-substrate junction organization, and performs the function of extracellular matrix binding, fibronectin binding and calcium-dependent phospholipid binding. It regulates the pathway of focal adhesion, PI3K-Akt signaling pathway and leukocyte transendothelial migration. Myeloid_C9_DTL in the peritumor participates in the biological process of response to tumor necrosis factor, monocyte chemotaxis and mononuclear cell migration, and exerts the molecular function of CCR chemokine receptor binding, chemokine activity and cytokine activity. It regulates the pathway of Toll-like receptor signaling pathway, NF-kappa B signaling pathway and cytokine-cytokine receptor interaction (Fig. s2D). The protein–protein interaction networks of Myeloid_C9_DTL in the tumor and peritumor are shown in Figs. 5D, E. By tracing the developmental trajectory of the myeloid cell clusters, we can infer that Myeloid_C4_EGFR, Myeloid_C6 and Myeloid_C8 are potential differentiation endpoints of myeloid (Fig. 5F and Fig. 5G). Over time, during the differentiation process of the myeloid cell clusters, the expression levels of *CSMD2*, *GRIK3* and *KAZN* increase (Fig. s2E). Over time, the dynamic expression profile of the top 100 genes in the myeloid cell clusters can be observed in Fig. 5H. Myeloid cells with high expression of *TNFAIP8L3* towards the M1 phenotype, predominantly present in the peritumor, are involved in TNF-α signaling via NF-kB and inflammatory response. At the protein level, the core protein network associated with *TNFAIP8L3* contains chemokines (*CCL2*, *CCL3*, *CCL4* and *CCL5*), implying that myeloid cells with high expression of *TNFAIP8L3* can be recruited into the GBM microenvironment to exert anti-tumor effects. Myeloid cells with high expression of *DTL* towards the M2 phenotype are proliferative, slightly more abundant in tumors, and involved in the G2/M checkpoint and E2F targets-related pathways. At the protein level, VIM, TGFBI and ANXA2 interacted in the tumor. FOS, DUSP1, and EGR1 interacted in the peritumor. Myeloid cells with high expression of *DTL* may be involved in angiogenesis in GBM.

### The function of neuronal clusters in the GBM microenvironment

**Fig. 6:**
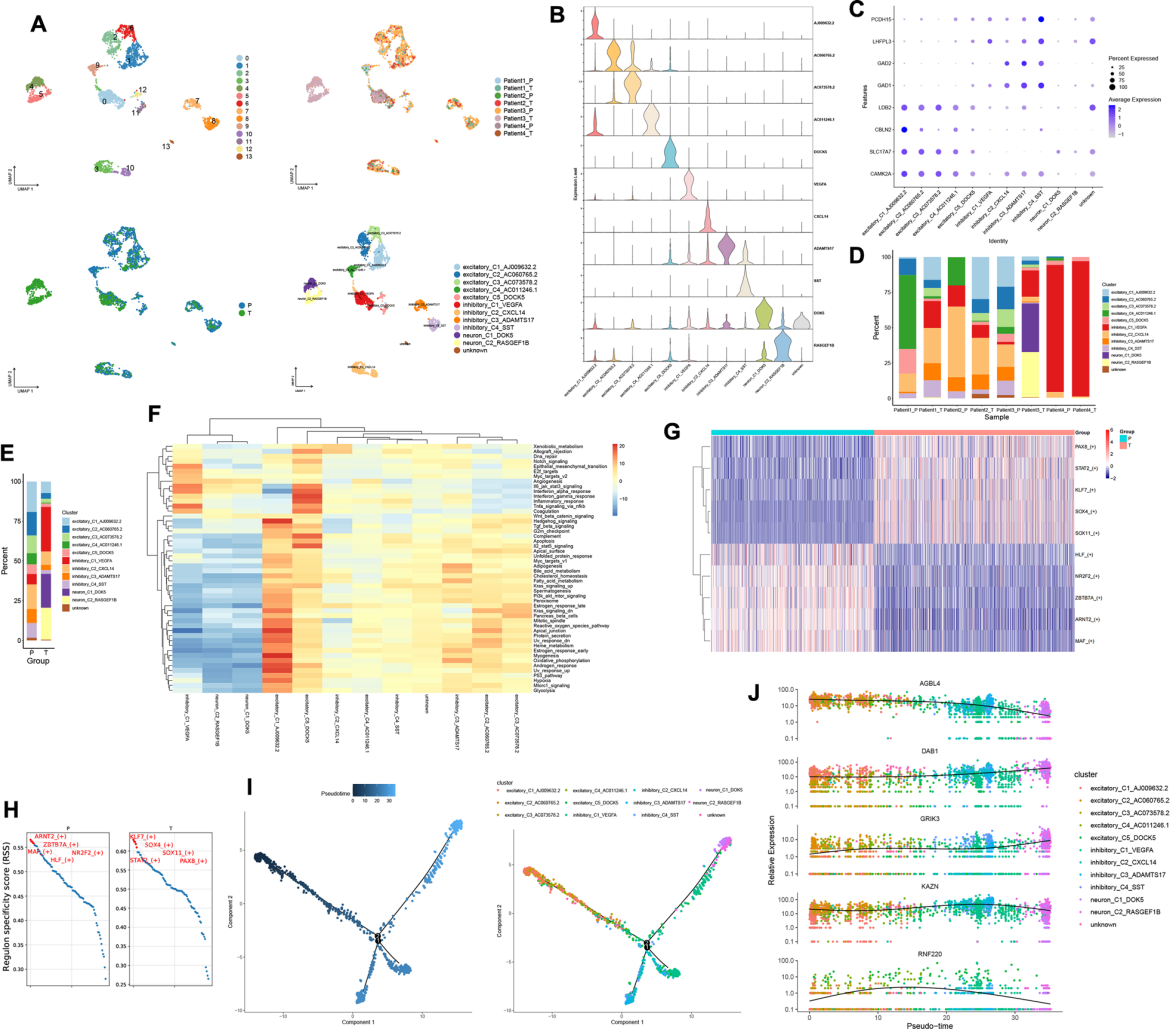
The function of neuronal clusters in the GBM microenvironment. **A** UMAP plots of neuronal cell clusters in the GBM microenvironment, 14 clusters (top left), patient origin (top right), site of origin (bottom left), annotated cell subgroups (bottom right). Each point represents a cell, with different colors indicating different cell clusters, patients, and origins. **B** One gene from the top 20 genes in each cell cluster is selected as a biomarker for that specific cell cluster. **C** Markers for excitatory and inhibitory neurons. **D** and **E** Percentage distribution of cell clusters after annotation, with different colors representing different cell clusters. **F** GSVA, a heatmap showing pathway enrichment for each cell cluster. **G** and **H** Top 5 transcription factors for both tumor and peritumor. **I** Cell trajectory inference, with each point representing a cell. The gradient from deep blue to light blue indicates time progression from early to late (left), and different colors represent different cell clusters (right). **J** Dynamic expression of top 5 genes in neuronal cell clusters, with different colors representing different clusters

3126 neurons were divided into 14 clusters through unsupervised clustering, and their patients and site origins can be seen in Fig. 6A. We annotated these cell clusters based on previously reported markers and the top genes for each cluster (Fig. 6B, C and Table S1). These cell clusters were annotated as excitatory_C1_AJ009632.2, excitatory_C2_AC060765.2, excitatory_C3_AC073578.2, excitatory_C4_AC011246.1, excitatory_C5_DOCK5, inhibitory_C1_VEGFA, inhibitory_C2_CXCL14, inhibitory_C3_ADAMTS17, inhibitory_C4_SST, neuron_C1_DOK5 and neuron_C2_RASGEF1B (Fig. 6A). The Neuron cell clusters exhibit significant heterogeneity across patients. inhibitory_C1_VEGFA is primarily present in Patient 4. neuron_C1_DOK5 and neuron_C2_RASGEF1B are predominantly found in the tumor of Patient 3. excitatory_C4_AC011246.1 and excitatory_C5_DOCK5 are mainly present in the peritumoral tissue of Patient 1 (Fig. 6D). Excitatory neurons account for over 50% of the content in the peritumor, while inhibitory, non-excitable and non-inhibitory neurons overwhelmingly dominate the content within the tumor (Fig. 6E and Fig. s3B). Neuron cell clusters in the tumor mainly enriched pathways such as angiogenesis, IL6 Jak-STAT3 signaling and Interferon alpha response, while in the peritumor, they primarily enriched pathways like oxidative phosphorylation, Kras signaling up and fatty acid metabolism (Fig. s3A). excitatory_C5_DOCK5 is mainly enriched in pathways such as interferon alpha response, interferon gamma response and complement. inhibitory_C1_VEGFA primarily enriches pathways including TNF-α signaling via NF-kB, interferon alpha response and IL6 Jak-STAT3 signaling (Fig. 6F). In the tumor, the top 5 transcription factors for neuron cell clusters are *KLF7*, *SOX4*, *SOX11*, *STAT2* and *PAX8*. Meanwhile, in the peritumor, the top 5 transcription factors for neuron cell clusters are *ARNT2*, *ZBTB7A*, *MAF*, *NR2F2* and *HLF* (Fig. 6G, H). These active transcription factors in excitatory neurons are *ATF6*, and in inhibitory_C1_VEGFA, the active transcription factor is *ATF4* (Fig. s3C). neuron_C1_DOK5 and neuron_C2_RASGEF1B may represent terminal cell populations in the differentiation of neurons. The majority of excitatory neurons serve as the developmental starting point, transitioning gradually into inhibitory neurons, and ultimately leading to neuron_C1_DOK5 and neuron_C2_RASGEF1B (Fig. 6I). As time progresses, the expression of *AGBL4* in neurons gradually decreases, while the expression of *DAB1* and *GRIK3* increases (Fig. 6J). As time progresses, the dynamic expression of the top 100 genes during the neuronal differentiation process can be observed in supplementary Fig. 3D. Neuronal cells are highly heterogeneous among GBM patients, with different classes and functions of neuronal cells in different sites in different GBM patients.

### The role of malignant cell clusters in the GBM microenvironment

**Fig. 7:**
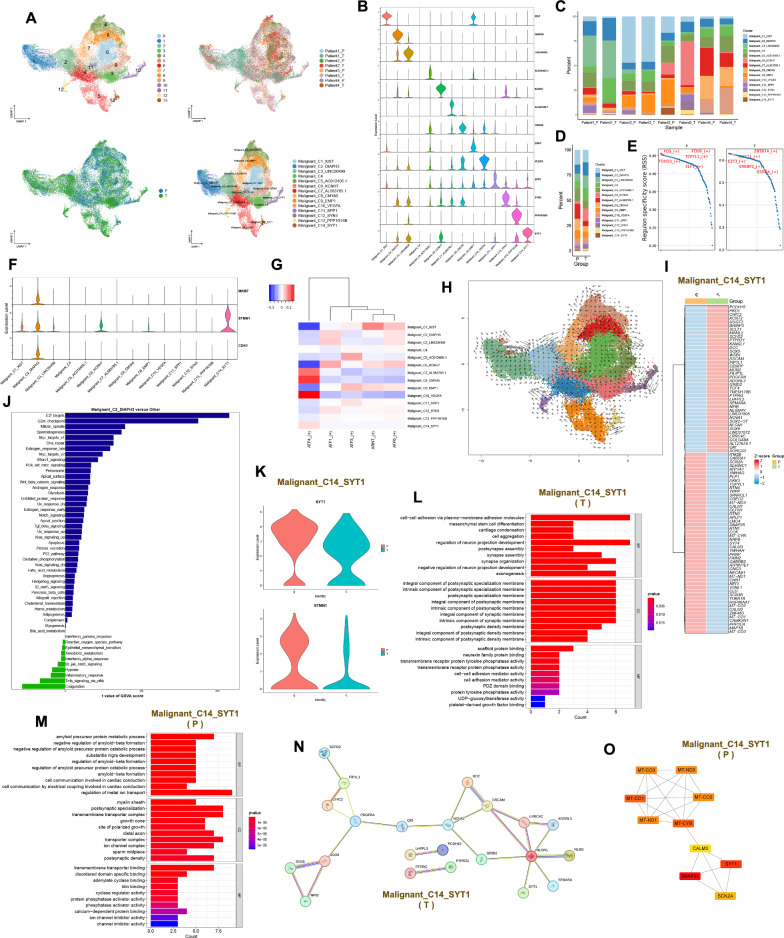
The role of malignant cell clusters in the GBM microenvironment. **A** UMAP plots of malignant cell clusters in the GBM microenvironment, 14 clusters (top left), patient origin (top right), site of origin (bottom left), annotated cell subgroups (bottom right). Each point represents a cell, with different colors indicating different cell clusters, patients and origins. **B** One gene from the top 20 genes in each cell cluster is selected as a biomarker for that specific cell cluster. **C** and **D** Percentage distribution of cell clusters after annotation, with different colors representing different cell clusters. **E** Top 5 transcription factors for both tumor and peritumor. **F** The expression of proliferative genes in each cell cluster. **G** SCENIC, a transcription factor heatmap for each cell cluster. **H** RNA velocity, where each point represents a cell, different colors indicate distinct cell clusters, arrow direction represents the potential differentiation trajectory of cells, and arrow size indicates the strength of differentiation ability. **I** Differential analysis in Malignant_C14_SYT1 using Wilcoxon test, with avg_log2FC > 0.4 and p_val_adj < 0.05. Red indicates high expression, while blue indicates low expression. **J** GSVA in Malignant_C2_DIAPH3 (Malignant_C2_DIAPH3 versus other cell clusters). **K** Expression levels of *SYT1* and *STMN1* in Malignant_C14_SYT1 in the tumor and peritumor. (L and M) GO functional enrichment of Malignant_C14_SYT1 in the tumor and peritumor. **N** and **O** Protein–protein interaction networks of Malignant_C14_SYT1 in the tumor and peritumor

38,598 malignant cells were divided into 14 clusters, and their patients and locations of origin can be seen in Fig. 7A. These malignant cell clusters were annotated as Malignant_C1_XIST, Malignant_C2_DIAPH3, Malignant_C3_LINC00499, Malignant_C4, Malignant_C5_AC012405.1, Malignant_C6_KCNH7, Malignant_C7_AL583785.1, Malignant_C8_CMYA5, Malignant_C9_EMP1, Malignant_C10_VEGFA, Malignant_C11_SPP1, Malignant_C12_SYN3, Malignant_C13_PPP1R16B and Malignant_C14_SYT1 based on the top 20 genes of each malignant cell cluster (Fig. 7A, B and Table S1). There is a large heterogeneity of malignant cell clusters across patients. Malignant_C3_LINC00499 is basically present in Patient 1. Malignant_C1_XIST is basically present in Patient 2. Malignant_C6_KCNH7, Malignant_C12_SYN3 and Malignant_C14_SYT1 are basically present in Patient 3. Malignant_C7_AL583785.1, Malignant_C8_CMYA5 and Malignant_C10_VEGFA are predominantly present in Patient 4 (Fig. 7C). The content of Malignant_C5_AC012405.1, Malignant_C7_AL583785.1 and Malignant_C8_CMYA5 is higher in the peritumor than in the tumor. Conversely, Malignant_C2_DIAPH3, Malignant_C4 and Malignant_C6_KCNH7 show higher content in the tumor than in the peritumor (Fig. 7D and Fig. s3E). The top 5 transcription factors active in the tumor for the malignant cell clusters are *ZBTB7A*, *SOX11*, *E2F3*, *SREBF2* and *ESRRA*. The top 5 transcription factors active in the peritumor for the malignant cell clusters are *FOS*, *FOSB*, *TCF7L1*, *FOXO3* and *ELF1* (Fig. 7E and Fig. s3F). The active transcription factor for Malignant_C8_CMYA5 and Malignant_C10_VEGFA is *ATF4*, while the active transcription factor for Malignant_C1_XIST is *ARNT* (Fig. 7G). Malignant_C1_XIST, Malignant_C6_KCNH7 and Malignant_C14_SYT1 highly express *STMN1*. Malignant_C2_DIAPH3 highly expresses *MKI67*, *STMN1* and *CDK1*. These results suggest that these four malignant cell clusters may have high proliferative potential (Fig. 7F). The cell developmental trajectories that we inferred by monocle do not elucidate well the developmental start and end points of these malignant cell clusters. Therefore, we have described the developmental direction of these malignant cell clusters by RNA velocity (Fig. s3G and Fig. 7H). Differential genes for Malignant_C1_XIST, Malignant_C2_DIAPH3, Malignant_C6_KCNH7 and Malignant_C14_SYT1 in the tumor and peritumor can be seen in supplementary Figs. 3I, 4E and 4 J as well as Fig. 7I. Malignant_C1_XIST is predominantly enriched in pathways such as cholesterol homeostasis, angiogenesis and myc targets v1. Malignant_C2_DIAPH3 is mainly enriched in pathways including E2F targets, G2/M checkpoint and mitotic spindle. Malignant_C6_KCNH7 is primarily enriched in pathways such as Hedgehog signaling, Kras signaling dn and Wnt beta-catenin signaling. Malignant_C14_SYT1 is mainly enriched in pathways including coagulation, Kras signaling dn and reactive oxygen species pathway (Fig. 7J and Fig. s4A). Malignant_C1_XIST expresses both *XIST* and *STMN1* in the tumor and peritumor (Fig. s3H). Malignant_C1_XIST is involved in biological processes such as cytoplasmic translation, ribosomal small subunit assembly and rRNA processing within the tumor, exerting molecular functions related to structural constituent of ribosome, mRNA 5' − UTR binding and rRNA binding. Meanwhile, in the peritumor, it participates in biological processes such as sleep, circadian behavior and rhythmic behavior, playing roles in molecular functions associated with RNA polymerase II core promoter sequence-specific DNA binding, DNA-binding transcription activator activity and RNA polymerase II-specific (Fig. s4B). The protein interaction network of Malignant_C1_XIST in the tumor and peritumor can be seen in supplementary Fig. 4C. Malignant_C2_DIAPH3 expresses *CDK1*, *DIAPH3*, *MKI67* and *STMN1* in both the tumor and the peritumor (Fig. s4D). In the tumor, Malignant_C2_DIAPH3 primarily participates in biological processes such as cell–cell adhesion via plasma-membrane adhesion molecules, positive regulation of cell projection organization and positive regulation of neuron projection development. Meanwhile, in the peritumor, it is actively involved in biological processes including vascular-associated smooth muscle cell development, negative regulation of axon regeneration and negative regulation of neuron projection regeneration (Fig. s4F and Fig. s4H). Malignant_C2_DIAPH3 was predicted to be present only in tumors with *NEGR1* and *LRRC7* protein–protein interactions (Fig. s4G). Malignant_C6_KCNH7 expresses *STMN1* and *KCNH7* in the tumor and peritumor (Fig. s4I). Malignant_C6_KCNH7 in the tumor is involved in biological processes such as regulation of postsynaptic membrane potential and regulation of membrane potential. However, in the peritumor, it participates in biological processes such as T cell differentiation, regulation of leukocyte differentiation and axon extension involved in axon guidance (Fig. s5A and Fig. s5B). Protein interaction networks of Malignant_C6_KCNH7 in the tumor and peritumor can be seen in supplementary Fig. 4 K. Malignant_C14_SYT1 expresses *STMN1* and *SYT1* in the tumor and peritumor (Fig. 7K). Malignant_C14_SYT1 in the tumor primarily participates in biological processes such as cell–cell adhesion via plasma-membrane adhesion molecules, cell aggregation and regulation of neuron projection development. Meanwhile, in the peritumor, it is mainly involved in processes such as regulation of metal ion transport, amyloid precursor protein metabolic process and negative regulation of amyloid-beta formation (Fig. 7L and Fig. 7M). Protein interaction networks of Malignant_C14_SYT1 in the tumor and peritumor can be seen in Figs. 7N, O. Malignant cell clusters with high expression of XIST, DIAPH3, KCNH7 and SYT1 have a strong proliferative capacity and they are present in the tumor and peritumor. Malignant cell clusters with high expression of XIST and DIAPH3 are found in all four patients. Malignant cell clusters with high XIST expression are associated with angiogenesis. At the protein level, the RPS gene family interacts with the RPL gene family in tumors, and FOS and EGR1 interact in the peritumor. DIAPH3 is mainly involved in malignant cell cycle regulation in the GBM microenvironment. At the protein level, LRRC7 interacts with NEGR1 in tumors. Malignant cell clusters with high expression of KCNH7 and SYT1 are mainly present in Patient 3 and are co-engaged in the Kras signaling pathway. At the protein level, SYT1 interacts with NLGN1 in the tumor, while SYT1, CALM3, SNAP25 and SCN2A interact in the peritumor. The SYT1 protein is also found to interact with the STMN2 protein in the tumor in the high-expressing KCNH7 malignant cell cluster.

### Intercellular communication

**Fig. 8:**
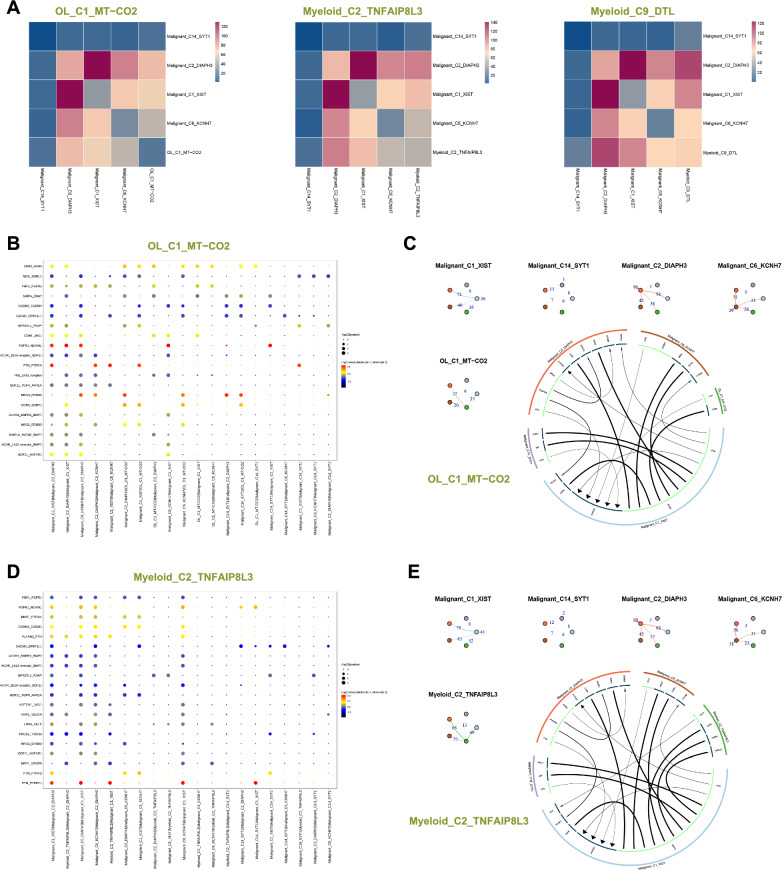
Intercellular communication. **A** Interactions heatmap between cell clusters, where the color gradient from blue to red represents an increasing number of interactions. **B** and **D** Interactions between two cell clusters via ligand-receptor pairs, where larger points indicate smaller p-values. The color gradient from dark gray to red represents the average expression level of ligand-receptor pairs from low to high. **C** and **E** Nodes represent cell clusters, nodes of the same color indicate the starting point, and reaching another node represents the endpoint. The numbers on the edges represent the number of ligand-receptor pairs, with thicker lines indicating more pairs (top left). The outer circle represents cell clusters, and the inner circle represents ligands or receptors. Arrows indicate direction, with the thickness of the lines representing the expression levels of the originating genes. The arrow size represents the expression levels of the recipient genes. Light green indicates the originating direction, while dark green represents the receiving direction (bottom right)

We selected OL_C1_MT-CO2 with high proliferation and high expression of mitochondria gene, Myeloid_C2_TNFAIP8L3 trending towards M1 type, Myeloid_C9_DTL with high proliferation trending towards M2 type, and proliferative malignant cell clusters (Malignant_C1_XIST, Malignant_C2_DIAPH3, Malignant_C6_KCNH7 and Malignant_C14_SYT1) to investigate their interactions in the TME. OL_C1_MT-CO2 and Myeloid_C2_TNFAIP8L3 have a high number of interactions with malignant cell clusters (Malignant_C1_XIST and Malignant_C2_DIAPH3). Myeloid_C9_DTL has a stronger interaction with malignant cell clusters (Malignant_C1_XIST, Malignant_C2_DIAPH3 and Malignant_C6_KCNH7) (Fig. 8A). Malignant_C2_DIAPH3 has a high number of interactions with these cell clusters (OL_C1_MT-CO2, Myeloid_C2_TNFAIP8L3 and Myeloid_C9_DTL) (Fig. 8C, E and Fig. s5D). We inferred intercellular communication using cellphonedb, revealing close interactions between Malignant_C2_DIAPH3 and OL_C1_MT-CO2 through *NRG3*-*ERBB4* and *COPA*-*SORT1*. Additionally, Malignant_C1_XIST shows close communication with OL_C1_MT-CO2 through *PTN*-*PTPRS* and *COPA*-*SORT1*. The malignant cell clusters (Malignant_C6_KCNH7 and Malignant_C14_SYT1) exhibit strong interactions with OL_C1_MT-CO2 through *NRG3*-*ERBB4* and *COPA*-*SORT1* (Fig. 8B). We further predicted intercellular communication networks using iTALK, revealing an interaction between *PTN* of OL_C1_MT-CO2 and *PTPRZ1* of Malignant_C1_XIST. Additionally, the *EGFR* of Malignant_C1_XIST interacts with *COPA* of OL_C1_MT-CO2 (Fig. 8C). Myeloid_C2_TNFAIP8L3 exhibits close communication with Malignant_C2_DIAPH3 through *PLXNB2*-*PTN*, and Myeloid_C2_TNFAIP8L3 shows close communication with Malignant_C1_XIST through *PTN*-*PTPRZ1* and *PLXNB2*-*PTN* (Fig. 8D). The *CD74* of Myeloid_C2_TNFAIP8L3 interacts with *APP* of Malignant_C1_XIST, and the *EGFR* of Malignant_C1_XIST interacts with *COPA*, *TGFB1* and *HBEGF* of Myeloid_C2_TNFAIP8L3 (Fig. 8E). Myeloid_C9_DTL exhibits a strong interaction with the malignant cell clusters (Malignant_C1_XIST, Malignant_C2_DIAPH3 and Malignant_C6_KCNH7) through the *SPP1*- a9b1 complex (Fig. s5C). The CD74 of Myeloid_C9_DTL interacts with *APP* of Malignant_C1_XIST. Additionally, the *EGFR* of Malignant_C1_XIST interacts with *COPA* and *TGFB1* of Myeloid_C9_DTL (Fig. s5D).

## Discussion

Mitochondria-associated genes may affect the prognosis of GBM patients and could serve as potential therapeutic targets to improve patient survival [22]. For 10 × single-cell sequencing data, mitochondrial ratio, an important parameter for assessing data quality, is often used to filter low-quality data. If the mitochondrial ratio is high, it may indicate that cell death has occurred, and this data with a high mitochondrial ratio should be filtered out before data analysis. In this study, we retained data with mitochondrial proportions below 5% for subsequent analysis. We identified a cluster of oligodendrocytes that highly express mitochondrial genes and also highly express a proliferative gene. This oligodendrocyte cluster is involved in oxidative phosphorylation and fatty acid metabolism, and is present in both tumor and peritumor. In the tumor, it is involved in MHC-I class I antigen processing and presentation and the regulation of T-cell-mediated cytotoxicity.

In the GBM microenvironment, the majority of immune cells are predominantly myeloid, so the composition of myeloid cell clusters plays a crucial role in successful immunotherapy and radiotherapy. However, most myeloid cells play an immunosuppressive and pro-tumorigenic role [23]. Macrophages belong to the myeloid. It is widely believed that in tumors, M1-type macrophages play a pro-inflammatory role, initiating immune responses and inhibiting tumor progression. In contrast, M2-type macrophages participate in anti-inflammatory processes, tissue remodeling, and promote tumor proliferation, invasion and metastasis, exerting immunosuppressive effects [24]. In this study, we observed that within the GBM microenvironment, it is challenging to distinctly differentiate between M1 and M2 macrophages; the majority of myeloid cell clusters co-express markers associated with both M1 and M2 phenotypes. This phenomenon has been reported in other studies as well, where heterogeneous clusters of tumor-associated macrophages/microglia expressing both M1 and M2 markers were also identified in GBM [25–27]. Subsequently, we calculated M1 and M2 scores for the myeloid cell clusters to assess which clusters exhibited a stronger inclination towards M1 or M2 phenotypes. We observed that the majority of myeloid cell clusters leaned more towards the M2 phenotype. Myeloid cell clusters with high expression of *TNFAIP8L3* tend to have an M1 phenotype and are essentially present in the peritumor. They are primarily involved in leukocyte adhesion and activation. Myeloid cell clusters with high expression of *DTL* tended to have an M2 phenotype, were more proliferative, and were present in both tumor and peritumor. In the tumor they were involved in focal adhesion and leukocyte transendothelial migration, while in the peritumor they were involved in the Toll-like receptor and NF-kappa B signaling pathway.

In this study, we observed that malignant cell clusters with high expression of *XIST*, *KCNH7*, *SYT1* and *DIAPH3* exhibit strong proliferative capabilities. *XIST* can promote the proliferation and migration of GBM through the miR-133a/SOX4 [28]. *XIST* can also promote the migration of human brain microvascular endothelial cells and enhance the angiogenesis of GBM [29]. miRNA-363-3p overexpression inhibits proliferation, invasion and metastasis of thyroid cancer cells, which is reversed by *SYT1* overexpression [30]. Increased expression of *DIAPH3* in cervical cancer may be associated with proliferation of cervical cancer [31]. *DIAPH3* expression is also associated with low survival of osteosarcoma patients and can promote proliferation and metastasis of osteosarcoma [32]. However, no studies have reported that *KCNH7* is involved in tumor progression. We also found that proliferative oligodendrocyte clusters with high expression of mitochondrial genes interact with these proliferative malignant cell clusters through *COPA*-*SORT1*. Myeloid cell clusters with high expression of *TNFAIP8L3* interact with malignant cell clusters with high expression of *DIAPH3* and *XIST* via *PLXNB2*-*PTN*. Myeloid cell clusters with high expression of *DTL* interact with malignant cell clusters with high expression of *XIST*, *DIAPH3* and *KCNH7* via the *SPP1*-a9b1 complex. Cellular components of the GBM microenvironment are altered by blocking or enhancing the interaction of these ligand-receptor pairs. These ligand-receptor pairs may be able to serve as novel therapeutic targets to prolong patient survival.

The effective treatment of GBM remains a challenge, mainly due to the low permeability and high selectivity of the blood–brain barrier towards traditional anti-cancer drugs. In recent years, nanomaterials have made significant progress in the field of GBM therapy as carriers for targeted delivery and more controllable drug release [33–35]. However, their application in clinical practice still faces enormous challenges. Our research helps to find more reliable therapeutic targets. Saliva contains electrolytes, nucleic acids, proteins, and microorganisms. Salivary omics can be used to discover tumor diagnostic and therapeutic markers [36]. Compared to solid tumors, saliva is easily accessible and non-invasive. Combining salivary omics with single-cell omics to explore the correlation of biomarkers between saliva and solid tumors, and applying the discovered salivary biomarkers to clinical application, will reduce the cost of diagnosis and treatment for patients. In our study, functional enrichment analysis revealed enrichment of microbiota-related pathways in GBM, implying that microbes may be present in the GBM microenvironment. Chalcone compounds, as derivatives of carbonyl compounds, possess antibacterial activity [37]. Chalcone compounds may be relevant to GBM treatment, further exploration through animal and clinical experiments is needed.

In conclusion, we characterized the cellular map of the GBM microenvironment by 10 × single-cell sequencing, revealing differences in the distribution and function of tumor and peritumor cells. We found strong heterogeneity in subpopulations of oligodendrocytes, myeloid, neurons and malignant cells across patients. We also identified a proliferative oligodendrocyte cluster with high expression of mitochondrial genes, a myeloid cell cluster tending towards an M1 phenotype with high expression of *TNFAIP8L3*, which are mainly present in the peritumor, and a proliferative myeloid cell cluster tending towards an M2 phenotype with high expression of *DTL*. *XIST*, *KCNH7*, *SYT1* and *DIAPH3* may be associated with the proliferation of malignant cells. These newly discovered cell clusters and markers may be available for clinical diagnosis and targeted therapy.

### Supplementary Information

Additional file 1: Figure S1. Data quality control and cell distribution. (A) The data distribution before quality control. (B) The data distribution after quality control for each patient. (C) Percentage distribution of 24 cell clusters, with different colors representing different cell clusters. (D) Differences in the content of cell clusters between the tumor and its surrounding area in oligodendrocytes (top) and myeloid (bottom). (E) GSVA in myeloid (tumor vs peritumor).

Additional file 2: Figure S2. Markers and functional enrichment of myeloid subtypes. (A and B) Differential analysis in Myeloid_C2_TNFAIP8L3 and Myeloid_C9_DTL, using Wilcoxon test, with avg_log2FC > 0.4 and p_val_adj < 0.05. Red indicates high expression, while blue indicates low expression. (C) Expression levels of M1 and M2 markers in different cell clusters. (D) GO (top) and KEGG (bottom) functional enrichment of Myeloid_C9_DTL in the tumor and peritumor. (E) Dynamic expression of top 5 genes in myeloid cell clusters, with different colors representing different clusters.

Additional file 3: Figure S3. Transcriptional regulation in neurons and malignant cells. (A) GSVA in neurons (tumor vs peritumor). (B) Differences in the content of cell clusters between the tumor and its surrounding area. (C) SCENIC, a transcription factor heatmap for each cell cluster. (D) Dynamic expression of top 100 genes in neuronal cell clusters, with a gradient from blue to red indicating expression levels from low to high. (E) Differences in the content of cell clusters between the tumor and its surrounding area. (F) Top 5 transcription factors for both tumor and peritumor in malignant cell clusters. (G) Cell trajectory inference, with each point representing a cell. The gradient from deep blue to light blue indicates time progression from early to late (left), and different colors represent different cell clusters (right). (H) Expression levels of *XIST* and *STMN1* in Malignant_C1_XIST in the tumor and peritumor. (I) Differential analysis in Malignant_C1_XIST using Wilcoxon test, with avg_log2FC > 0.4 and p_val_adj < 0.05. Red indicates high expression, while blue indicates low expression.

Additional file 4: Figure S4. Functional enrichment in malignant cell subtypes. (A) GSVA in Malignant_C1_XIST, Malignant_C6_KCNH7 and Malignant_C14_SYT1. (B) GO functional enrichment of Malignant_C1_XIST in the tumor and peritumor. (C) Protein-protein interaction networks of Malignant_C1_XIST in the tumor and peritumor. (D) Expression levels of *DIAPH3*, *STMN1*, *MKI67* and *CDK1* in Malignant_C2_DIAPH3 in the tumor and peritumor. (E) Differential analysis in Malignant_C2_DIAPH3 using Wilcoxon test, with avg_log2FC > 0.4 and p_val_adj < 0.05. Red indicates high expression, while blue indicates low expression. (F and H) GO functional enrichment of Malignant_C2_DIAPH3 in the tumor and peritumor. (G) Protein-protein interaction networks of Malignant_C2_DIAPH3 in the tumor. (I) Expression levels of *STMN1* and *KCNH7* in Malignant_C6_KCNH7 in the tumor and peritumor. (J) Differential analysis in Malignant_C6_KCNH7 using Wilcoxon test, with avg_log2FC > 0.4 and p_val_adj < 0.05. Red indicates high expression, while blue indicates low expression. (K) Protein-protein interaction networks of Malignant_C6_KCNH7 in the tumor and peritumor.

Additional file 5: Figure S5. Functional enrichment and intercellular communication. (A and B) GO functional enrichment of Malignant_C6_KCNH7 in the tumor and peritumor. (C) Interactions between two cell clusters via ligand-receptor pairs, where larger points indicate smaller p-values. The color gradient from dark gray to red represents the average expression level of ligand-receptor pairs from low to high. (D) Nodes represent cell clusters, nodes of the same color indicate the starting point, and reaching another node represents the endpoint. The numbers on the edges represent the number of ligand-receptor pairs, with thicker lines indicating more pairs (top left). The outer circle represents cell clusters, and the inner circle represents ligands or receptors. Arrows indicate direction, with the thickness of the lines representing the expression levels of the originating genes. The arrow size represents the expression levels of the recipient genes. Light green indicates the originating direction, while dark green represents the receiving direction (bottom right).

Additional file 6: Table S1. Top 20 genes in each cluster.

## Data Availability

Data in this study are available from the corresponding author upon reasonable request.
