# Supplementary figures and images for: Unveiling novel cell clusters and biomarkers in glioblastoma and its peritumoral microenvironment at the single-cell perspective
Source: J Transl Med. 2024 Jun 8;22:551. doi: 10.1186/s12967-024-05313-5 (PMC11162569; doi:10.1186/s12967-024-05313-5)

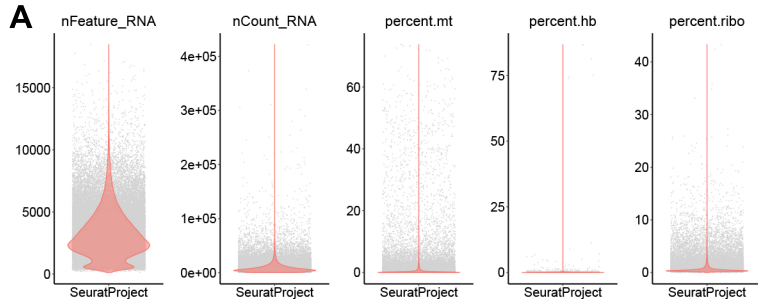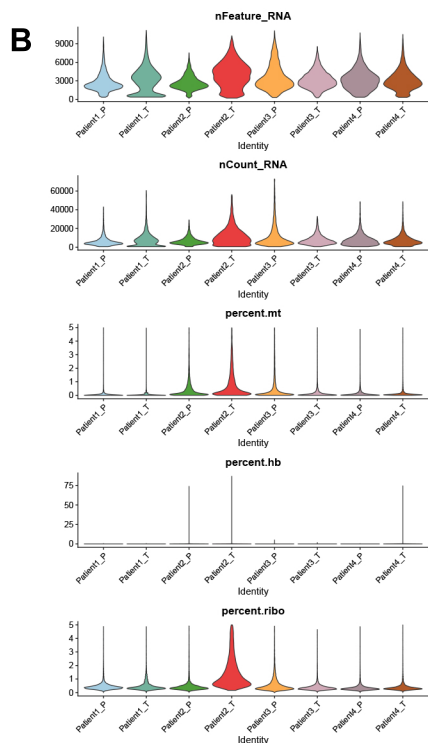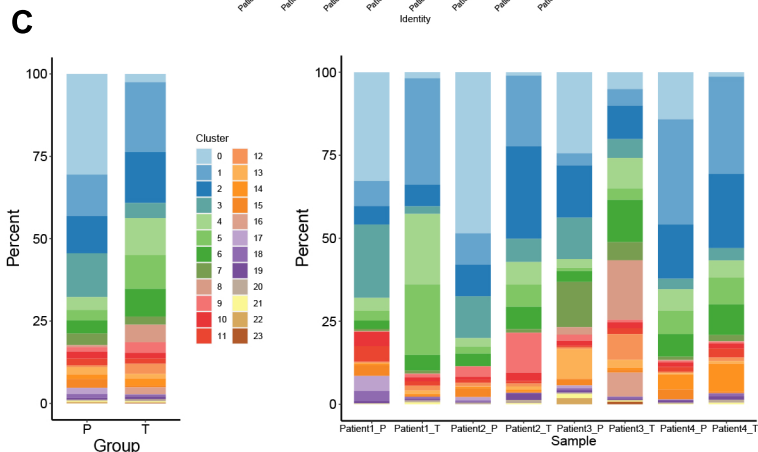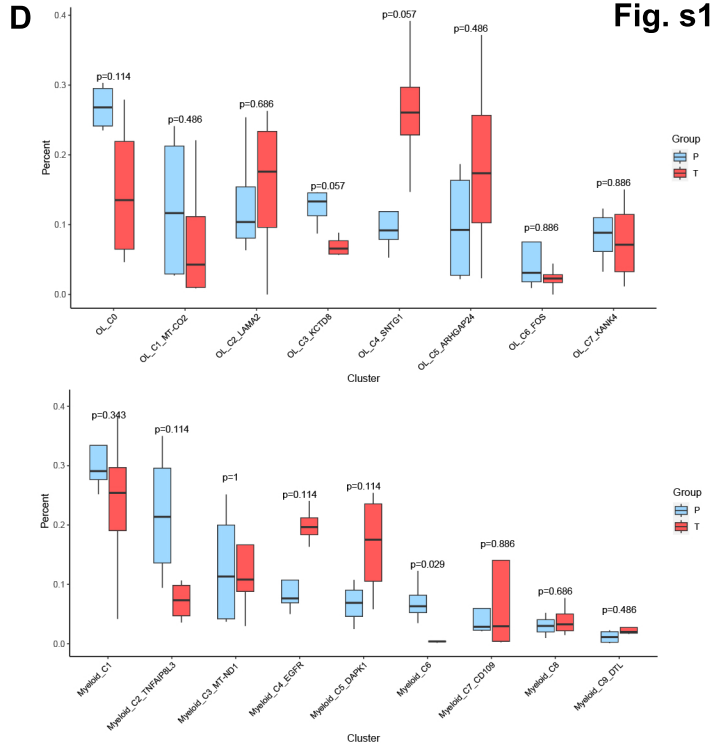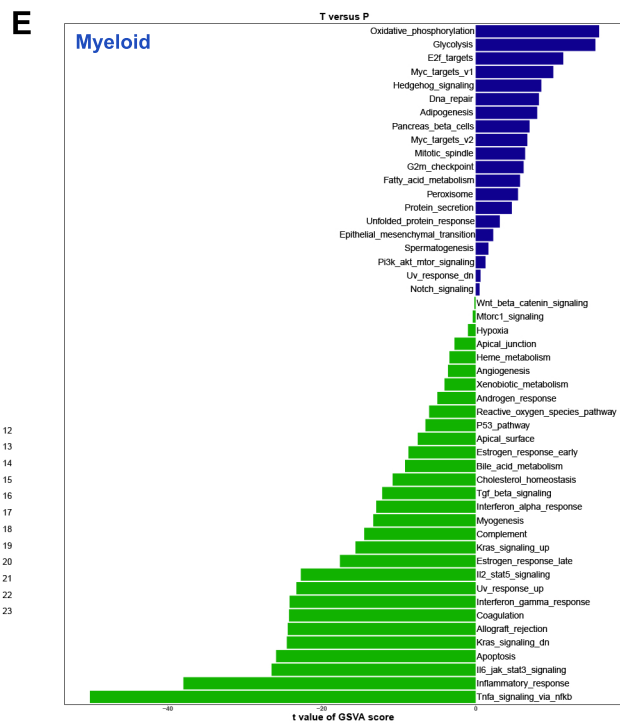

Supplement: Supplementary file 1 — Additional file 1: Figure S1. Data quality control and cell distribution. (A) The data distribution before quality control. (B) The data distribution after quality control for each patient. (C) Percentage distribution of 24 cell clusters, with different colors representing different cell clusters. (D) Differences in the content of cell clusters between the tumor and its surrounding area in oligodendrocytes (top) and myeloid (bottom). (E) GSVA in myeloid (tumor vs peritumor). [file 12967_2024_5313_MOESM1_ESM.pdf]

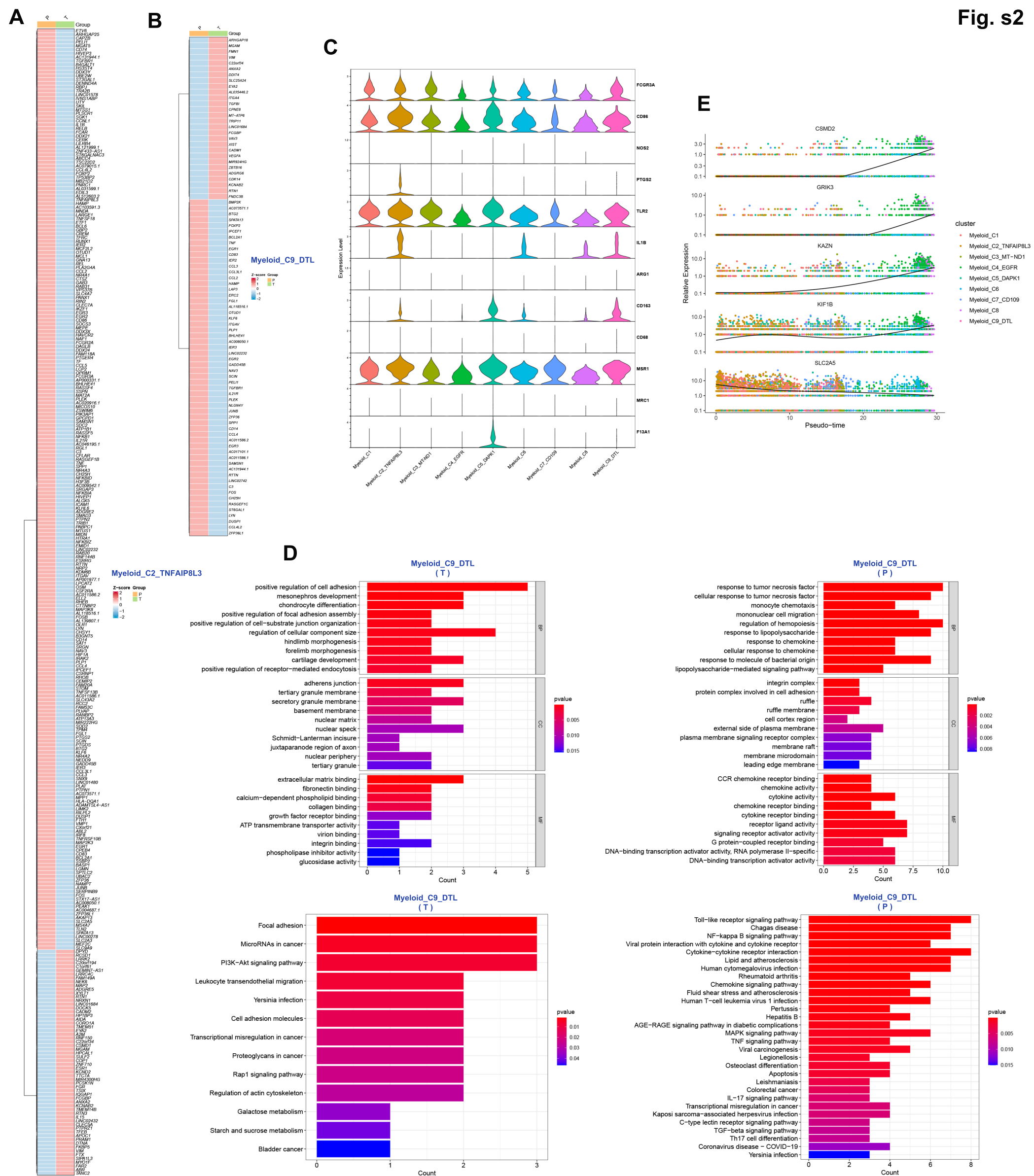

Supplement: Supplementary file 2 — Additional file 2: Figure S2. Markers and functional enrichment of myeloid subtypes. (A and B) Differential analysis in Myeloid_C2_TNFAIP8L3 and Myeloid_C9_DTL, using Wilcoxon test, with avg_log2FC > 0.4 and p_val_adj < 0.05. Red indicates high expression, while blue indicates low expression. (C) Expression levels of M1 and M2 markers in different cell clusters. (D) GO (top) and KEGG (bottom) functional enrichment of Myeloid_C9_DTL in the tumor and peritumor. (E) Dynamic expression of top 5 genes in myeloid cell clusters, with different colors representing different clusters. [file 12967_2024_5313_MOESM2_ESM.pdf]

**Fig. s3**

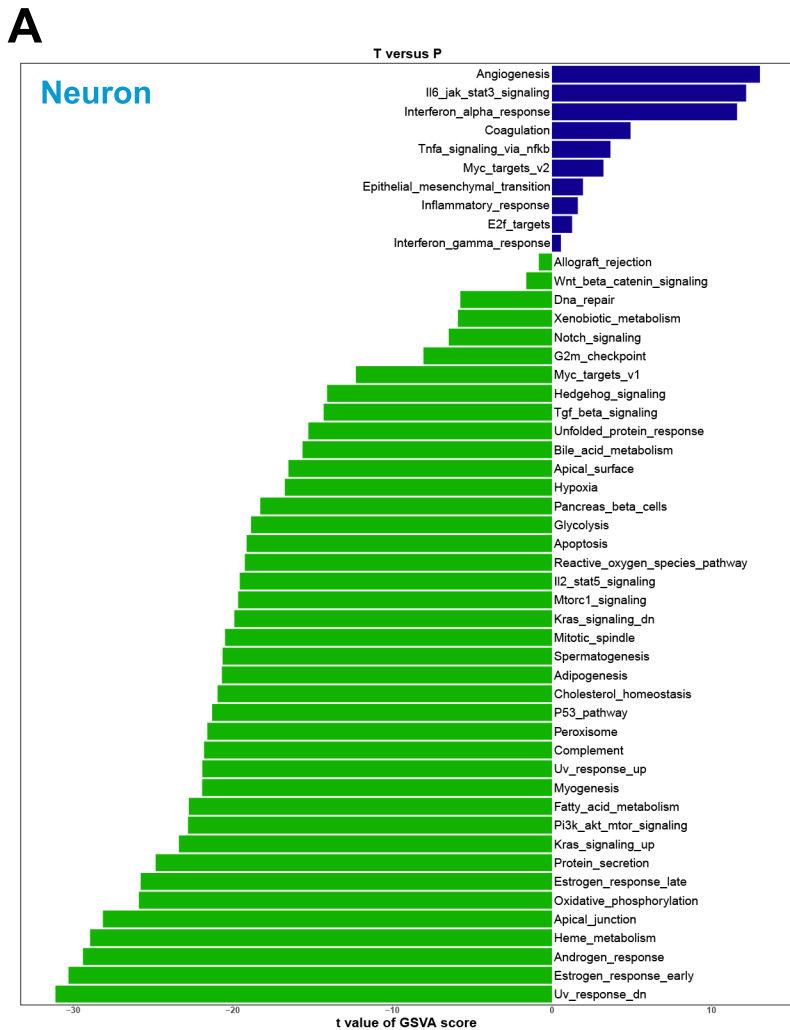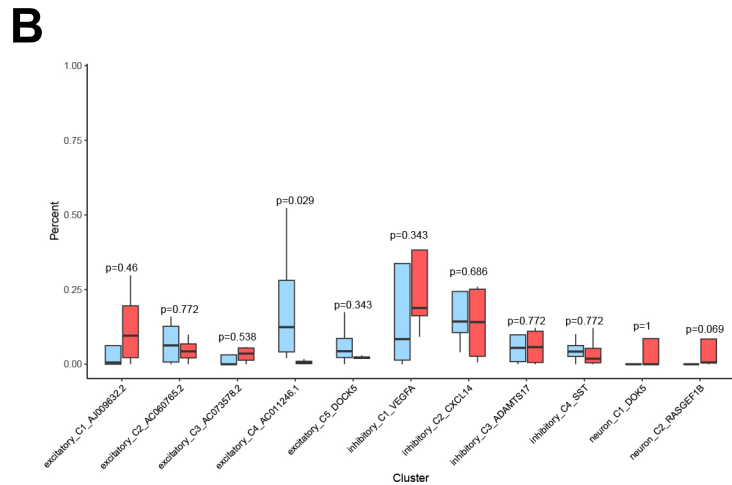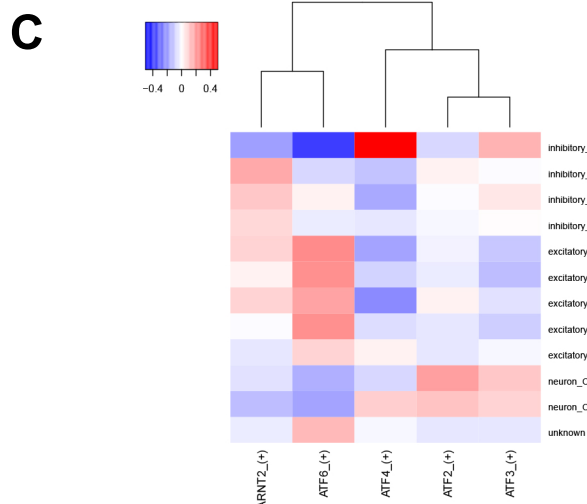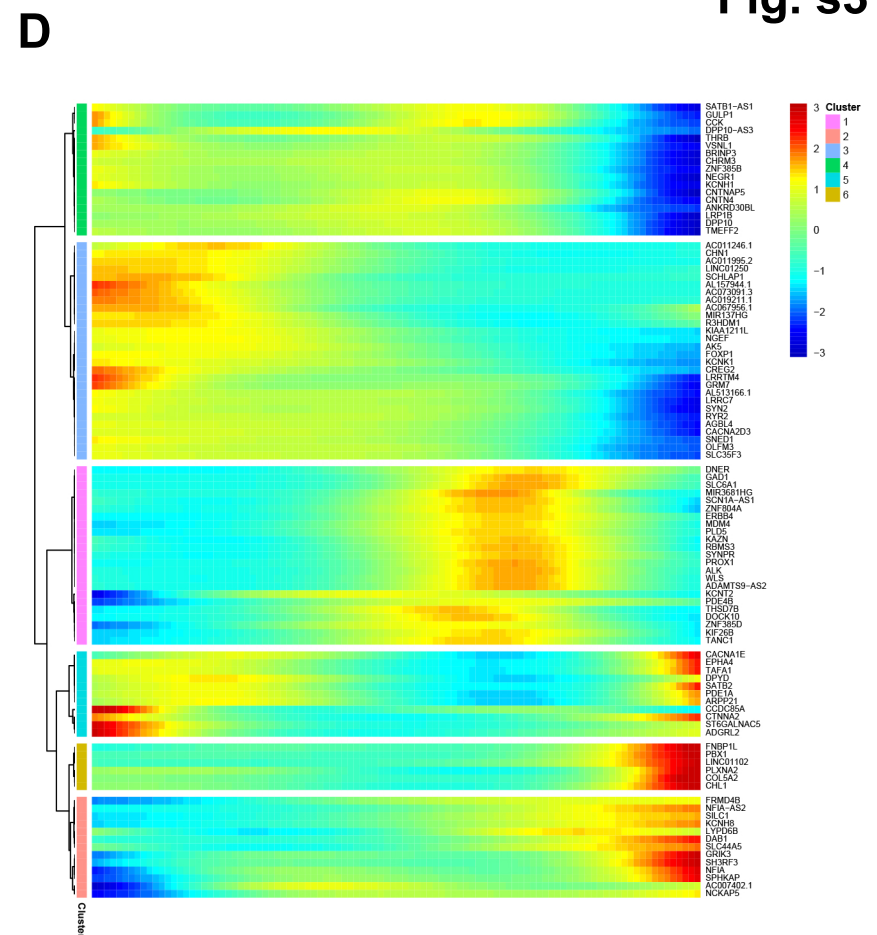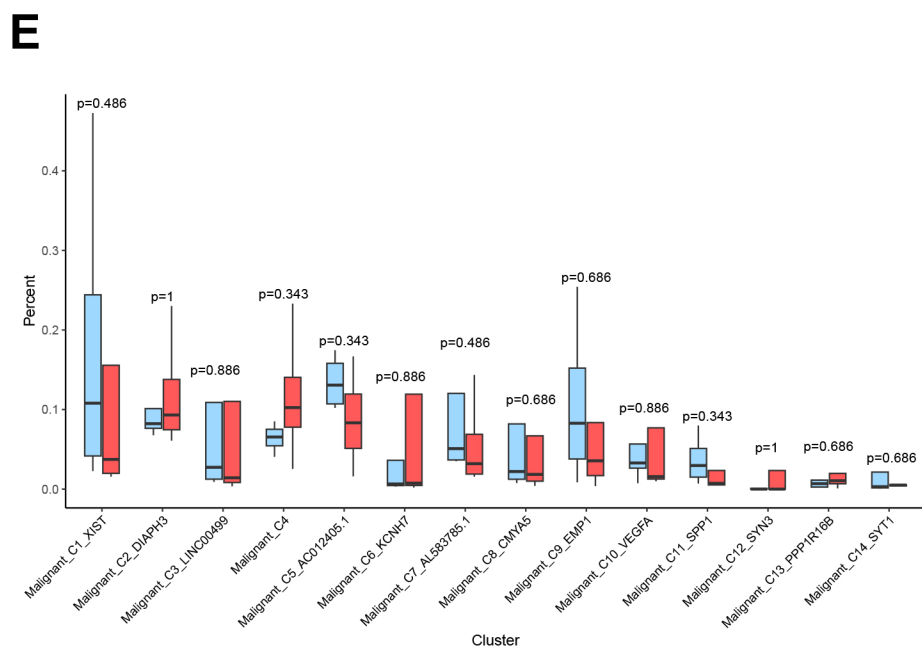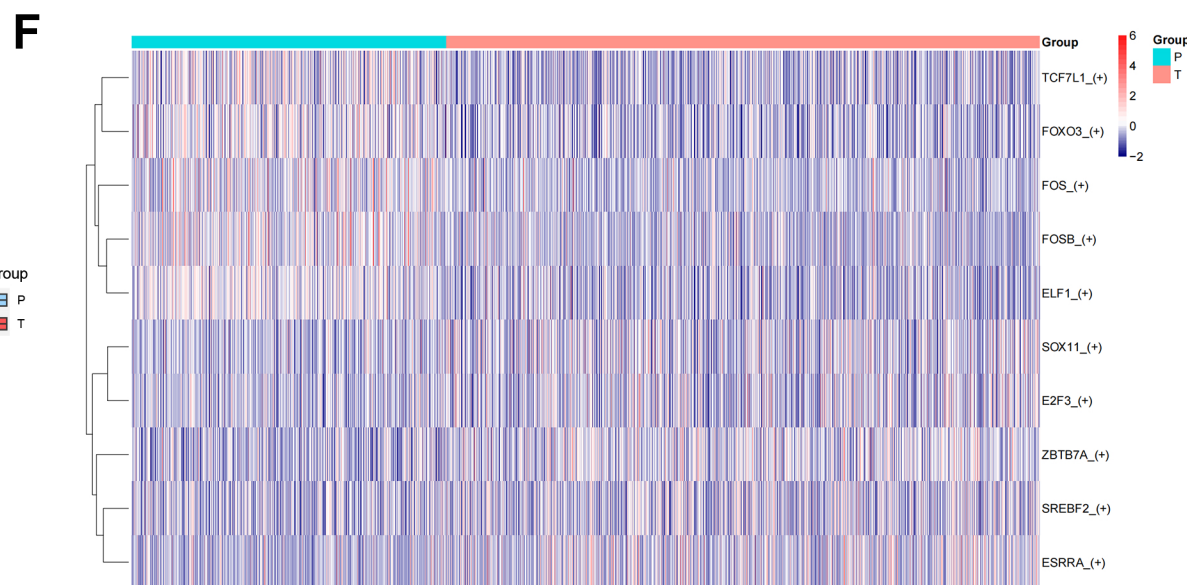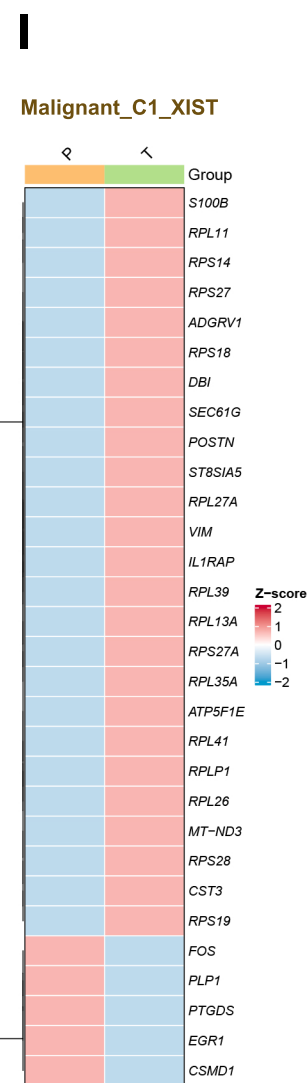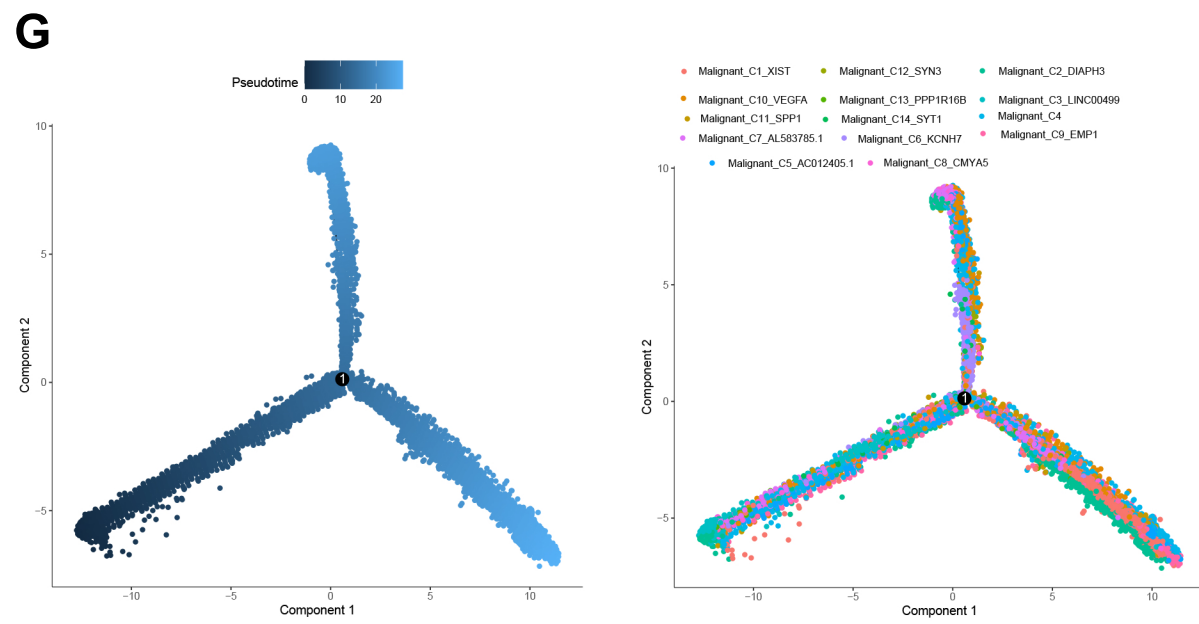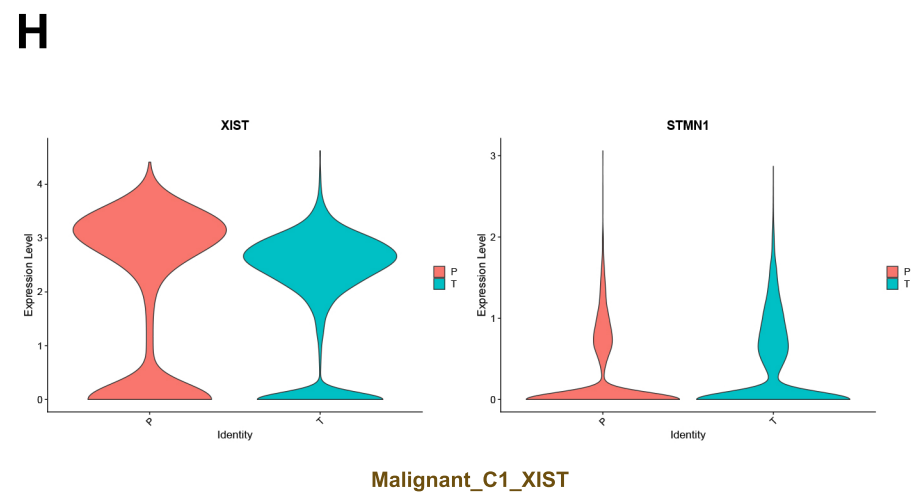

Supplement: Supplementary file 3 — Additional file 3: Figure S3. Transcriptional regulation in neurons and malignant cells. (A) GSVA in neurons (tumor vs peritumor). (B) Differences in the content of cell clusters between the tumor and its surrounding area. (C) SCENIC, a transcription factor heatmap for each cell cluster. (D) Dynamic expression of top 100 genes in neuronal cell clusters, with a gradient from blue to red indicating expression levels from low to high. (E) Differences in the content of cell clusters between the tumor and its surrounding area. (F) Top 5 transcription factors for both tumor and peritumor in malignant cell clusters. (G) Cell trajectory inference, with each point representing a cell. The gradient from deep blue to light blue indicates time progression from early to late (left), and different colors represent different cell clusters (right). (H) Expression levels of XIST and STMN1 in Malignant_C1_XIST in the tumor and peritumor. (I) Differential analysis in Malignant_C1_XIST using Wilcoxon test, with avg_log2FC > 0.4 and p_val_adj < 0.05. Red indicates high expression, while blue indicates low expression. [file 12967_2024_5313_MOESM3_ESM.pdf]

A

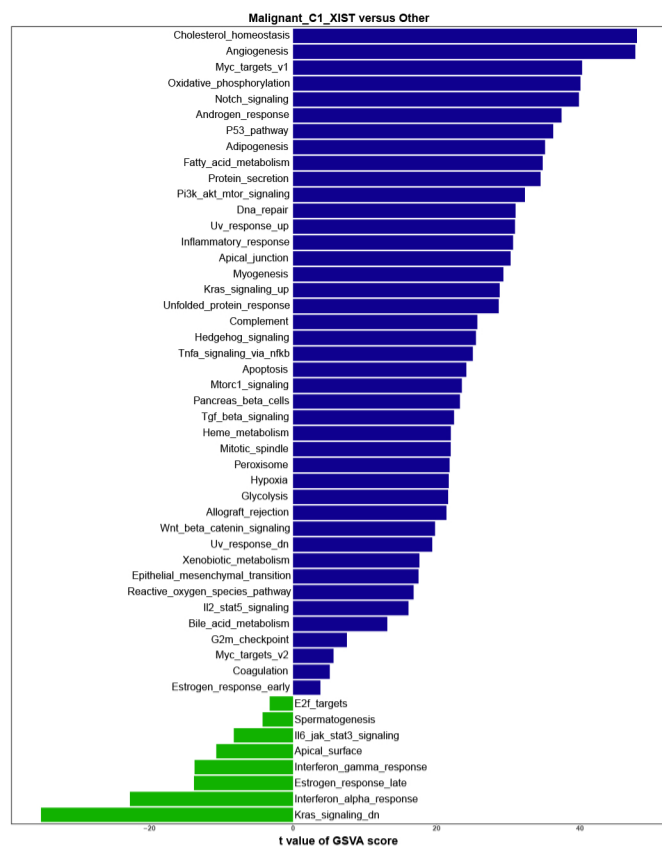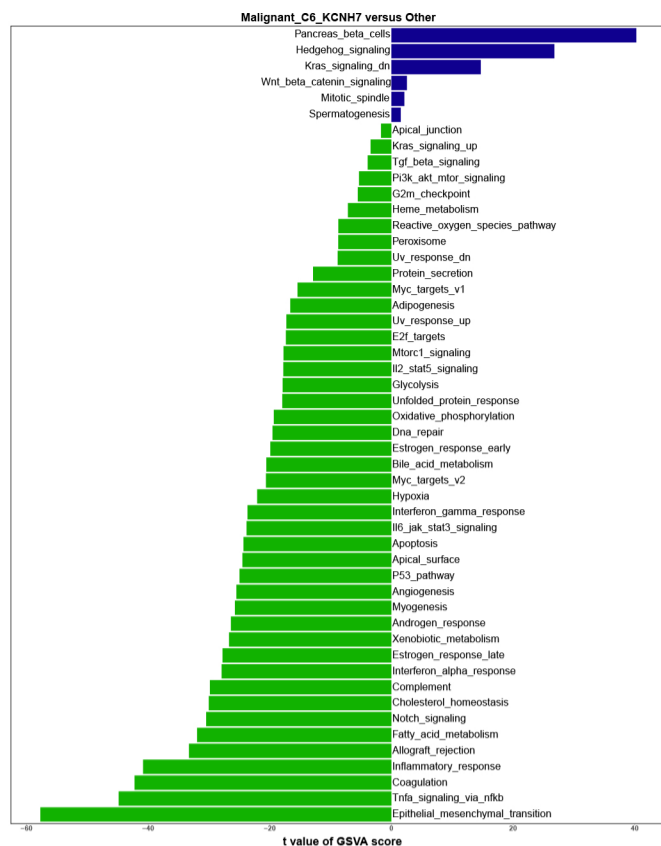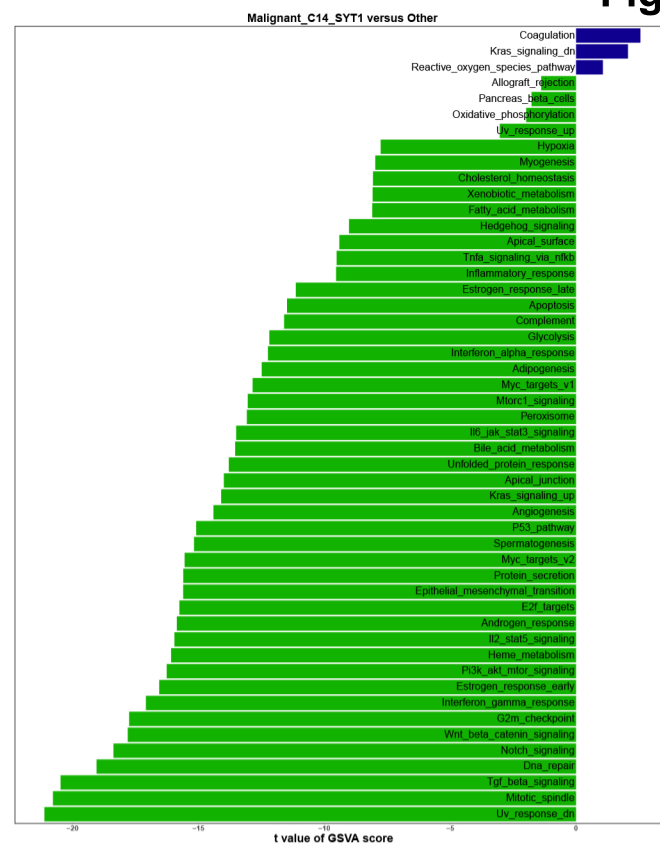

B

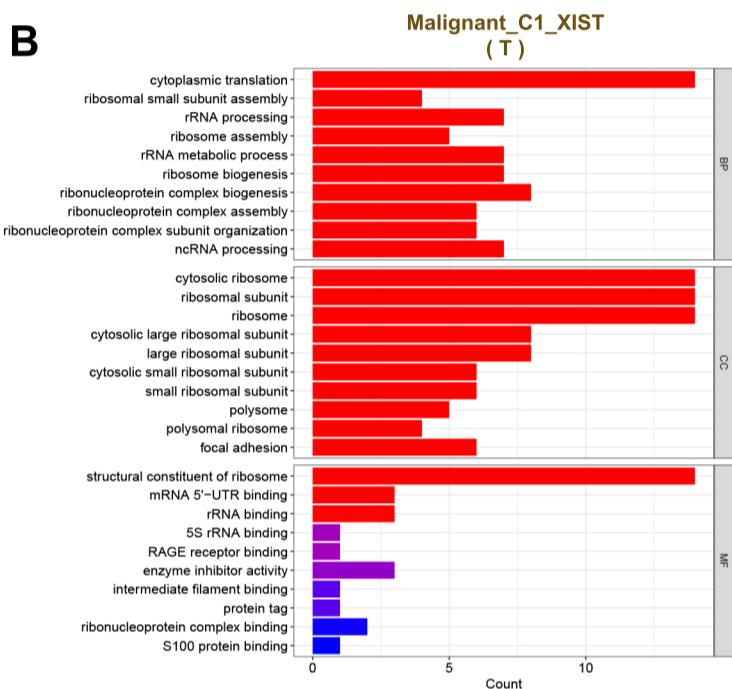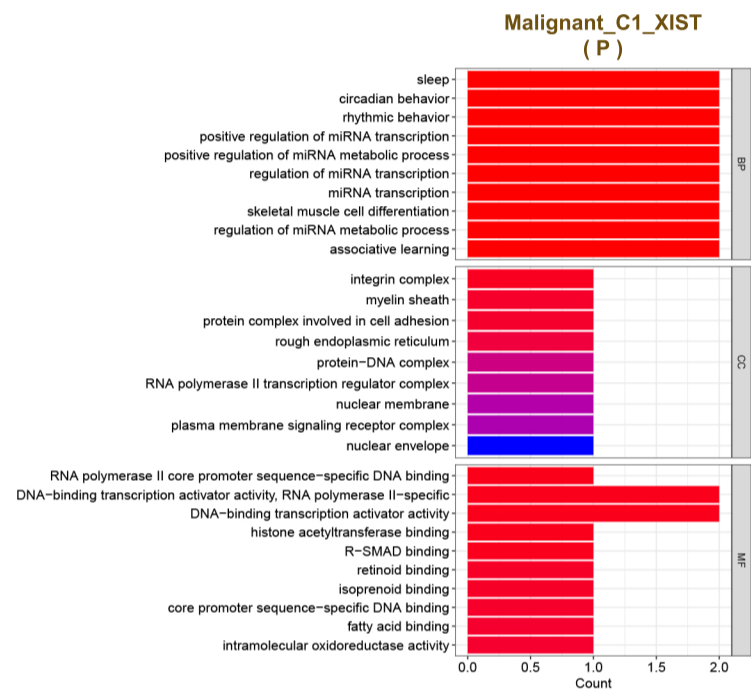

C

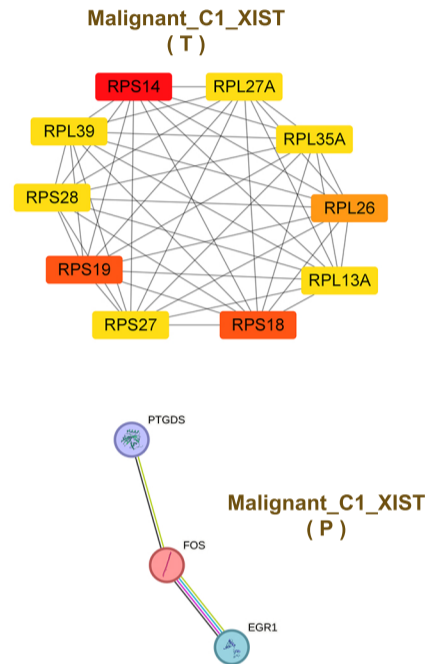

D

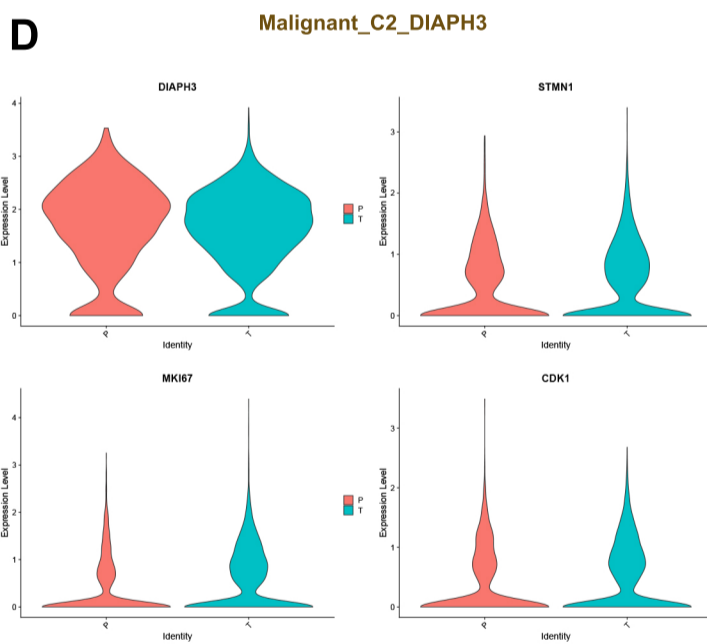

E

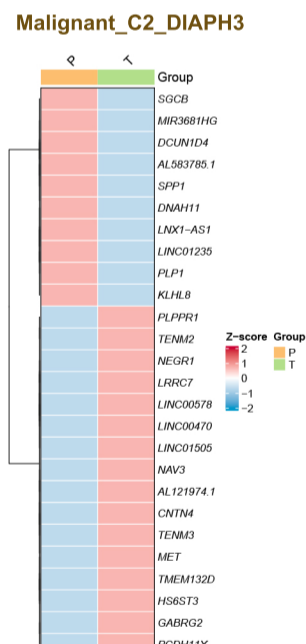

F

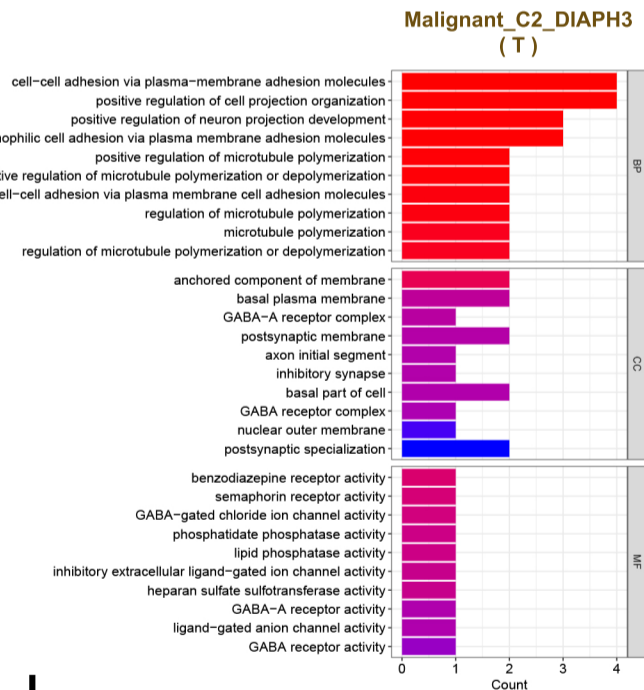

G

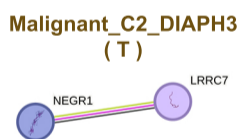

H

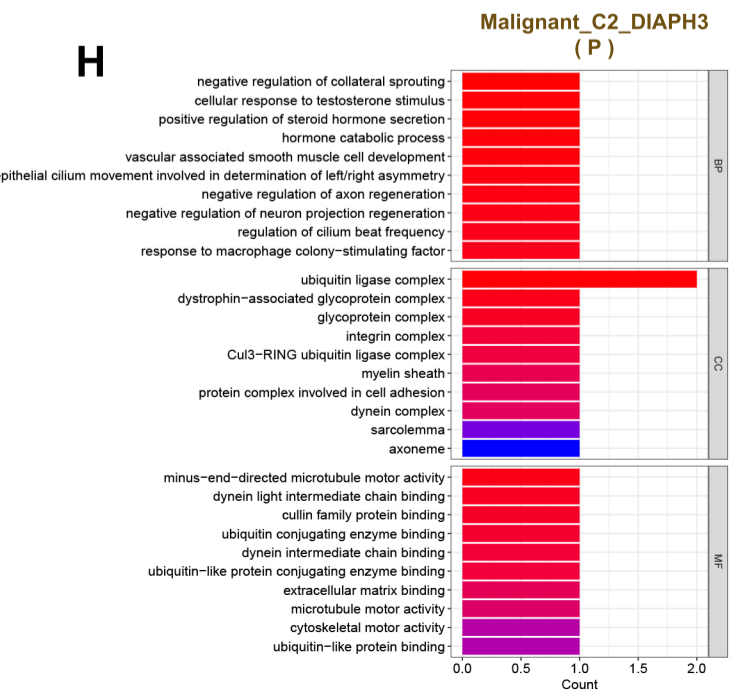

I

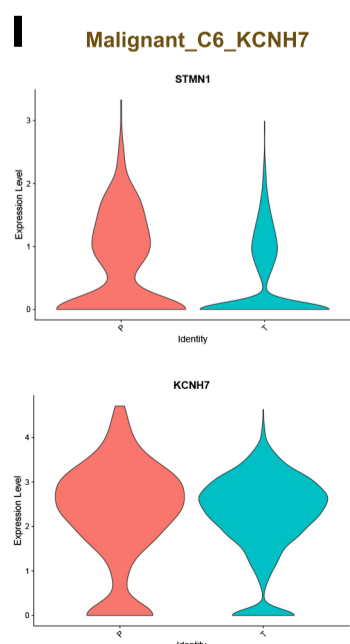

J

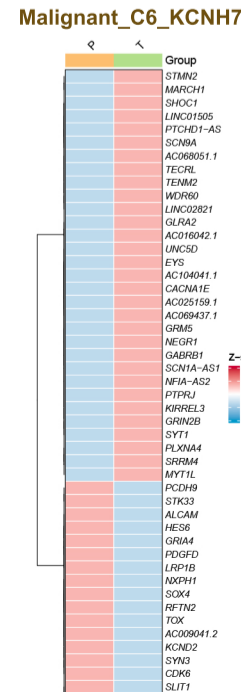

K

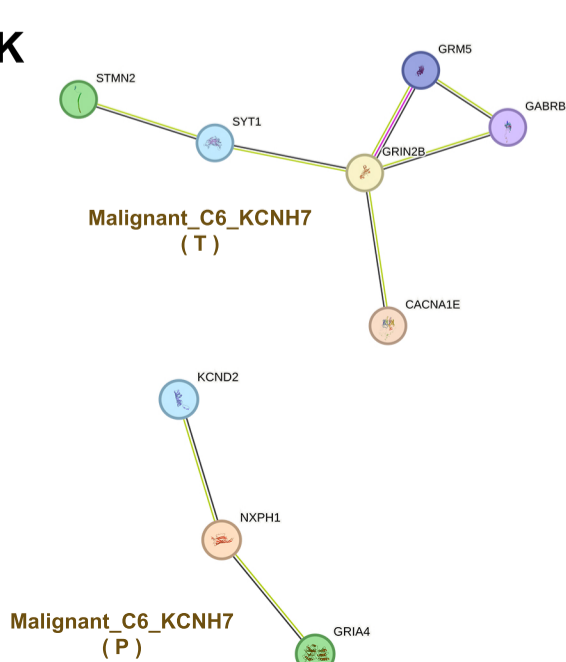

Supplement: Supplementary file 4 — Additional file 4: Figure S4. Functional enrichment in malignant cell subtypes. (A) GSVA in Malignant_C1_XIST, Malignant_C6_KCNH7 and Malignant_C14_SYT1. (B) GO functional enrichment of Malignant_C1_XIST in the tumor and peritumor. (C) Protein-protein interaction networks of Malignant_C1_XIST in the tumor and peritumor. (D) Expression levels of DIAPH3, STMN1, MKI67 and CDK1 in Malignant_C2_DIAPH3 in the tumor and peritumor. (E) Differential analysis in Malignant_C2_DIAPH3 using Wilcoxon test, with avg_log2FC > 0.4 and p_val_adj < 0.05. Red indicates high expression, while blue indicates low expression. (F and H) GO functional enrichment of Malignant_C2_DIAPH3 in the tumor and peritumor. (G) Protein-protein interaction networks of Malignant_C2_DIAPH3 in the tumor. (I) Expression levels of STMN1 and KCNH7 in Malignant_C6_KCNH7 in the tumor and peritumor. (J) Differential analysis in Malignant_C6_KCNH7 using Wilcoxon test, with avg_log2FC > 0.4 and p_val_adj < 0.05. Red indicates high expression, while blue indicates low expression. (K) Protein-protein interaction networks of Malignant_C6_KCNH7 in the tumor and peritumor. [file 12967_2024_5313_MOESM4_ESM.pdf]

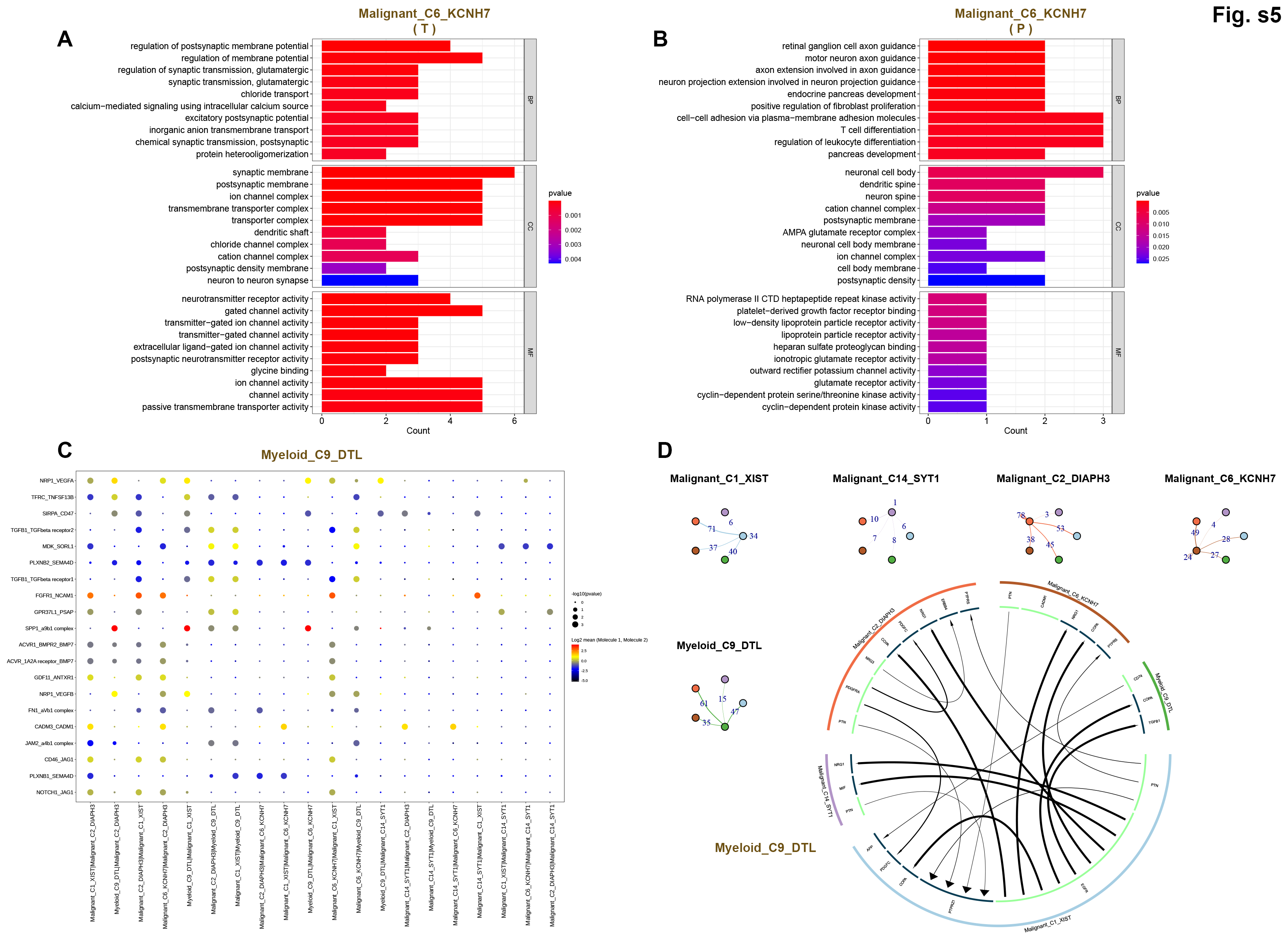

Supplement: Supplementary file 5 — Additional file 5: Figure S5. Functional enrichment and intercellular communication. (A and B) GO functional enrichment of Malignant_C6_KCNH7 in the tumor and peritumor. (C) Interactions between two cell clusters via ligand-receptor pairs, where larger points indicate smaller p-values. The color gradient from dark gray to red represents the average expression level of ligand-receptor pairs from low to high. (D) Nodes represent cell clusters, nodes of the same color indicate the starting point, and reaching another node represents the endpoint. The numbers on the edges represent the number of ligand-receptor pairs, with thicker lines indicating more pairs (top left). The outer circle represents cell clusters, and the inner circle represents ligands or receptors. Arrows indicate direction, with the thickness of the lines representing the expression levels of the originating genes. The arrow size represents the expression levels of the recipient genes. Light green indicates the originating direction, while dark green represents the receiving direction (bottom right). [file 12967_2024_5313_MOESM5_ESM.png]
